# Strong Nanomechanical Duffing Nonlinearity and Interactions Induced through Cavity Optomechanics

**DOI:** 10.1021/acs.nanolett.6c02784

**Published:** 2026-09-08

**Authors:** Jesse J. Slim, Ewold Verhagen

**Affiliations:** † Center for Nanophotonics, AMOLF, Science Park 104, 1098 XG Amsterdam, The Netherlands; ‡ Australian Research Council Centre of Excellence in Quantum Biotechnology (QUBIC), School of Mathematics and Physics, University of Queensland, St Lucia, Queensland 4072, Australia

## Abstract

Nonlinearity is a key resource in both classical and quantum signal processing. Nonlinear nanomechanical elements enable applications from sensing to computing, while networks of nonlinear or nonlinearly coupled resonators offer promising platforms for simulating complex dynamics. Here, we experimentally demonstrate an approach to realizing strong mechanical nonlinearity in nanomechanical resonators, which is fully controlled through optical laser drives. The mechanism exploits the intrinsic nonlinearity of the radiation pressure interaction in a cavity optomechanical system, giving rise to a nonlinear optical spring effect. The resulting Duffing nonlinearity is conveniently tunable in strength via spring laser power and in sign via detuning. Moreover, we demonstrate that the nonlinear optical spring mediates effective interactions between mechanical modes coupled to a common cavity, inducing tunable nonlinear interactions between them that impact the spectral response and dynamics. These results establish cavity optomechanics as a versatile and in situ reconfigurable platform for engineering nonlinear dynamics in resonators and networks.

## Graphical abstract

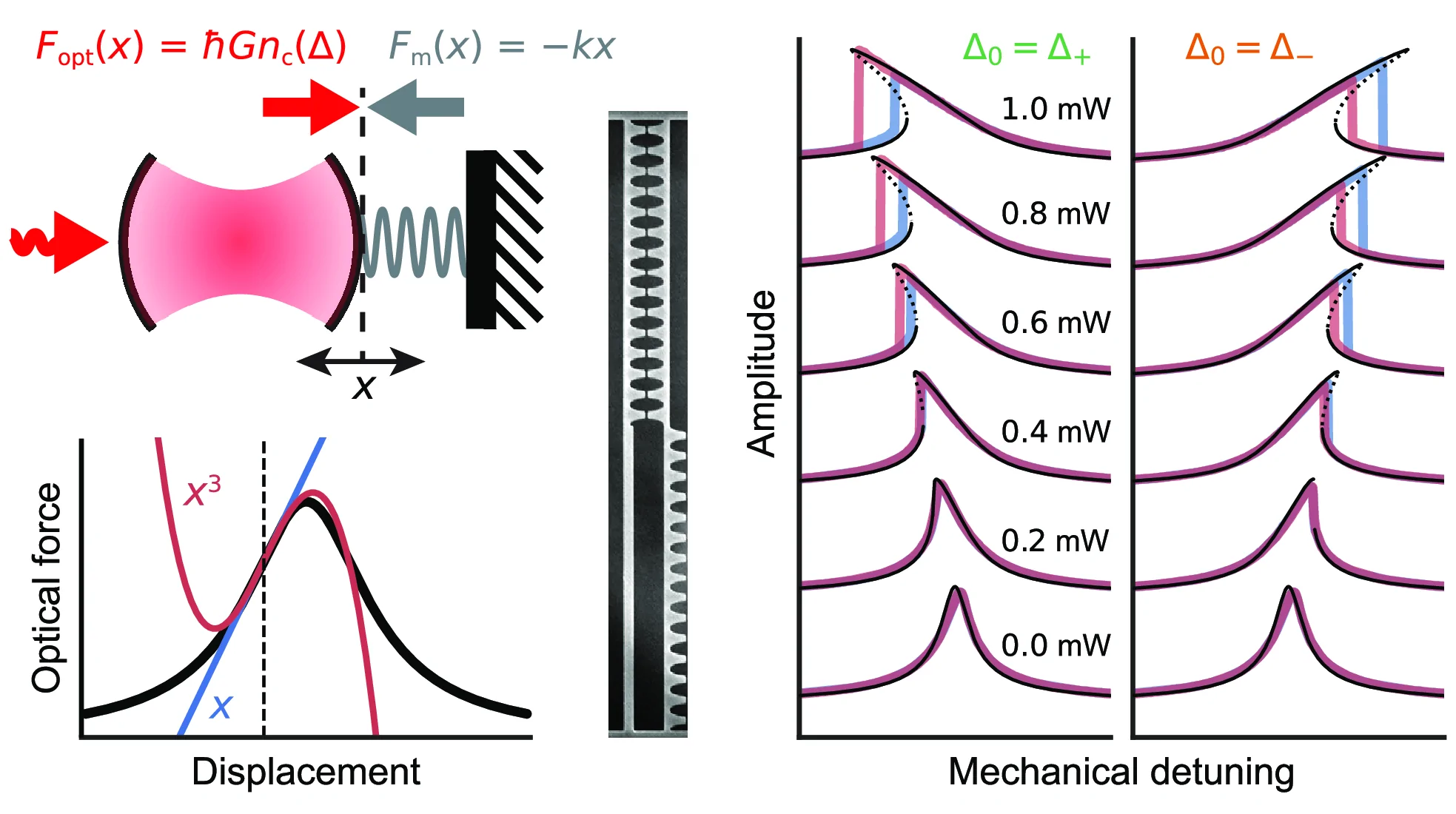

Nonlinearity is a pervasive and often decisive feature of nanomechanical resonators.^1^ Duffing-type dynamics give rise to phenomena such as frequency shifts, bistability, hysteresis, bifurcations, limit cycles, and chaos.^2–5^ It is also widely explored for applications in sensing, signal processing, and mechanical computing approaches.^6–8^ More broadly, exciting opportunities arise from controlled nonlinearity in both single resonators and networks or arrays: it can manipulate noise and fluctuations to yield synchronization,^9,10^ strong squeezing,^11^ and thermal engines;^12^ produce solitons and frequency combs;^13,14^ and enable novel computing paradigms including neuromorphic approaches and reservoir computing.^15–17^ In the quantum domain, nonlinearity enables quantum information processing and leads to intriguing correlated phases of matter.^18,19^ But also in classical arrays with nonlinear interactions fascinating phases of matter and collective dynamics have been predicted and observed, including nonlinear and dynamical topological phases^20–25^ and quantized nonlinear Thouless pumping.^26,27^ Specifically, networks with nonlinear interactions have attracted interest, for example for enabling topological solitons that could occur at self-induced traveling domain walls.^28–30^

Nanomechanics provides an excellent testbed for such dynamics as well as a promising technological application platform. However, even if nanomechanical nonlinearities of geometrical origin are ubiquitous, they are generally weak, requiring significant amplitudes on the order of the dimensions of a nanostructure (e.g., the thickness of a flexural beam). Moreover, as they are rooted in material properties and device geometry, they exhibit very limited *in situ* tunability. Finally, while electrostatic interactions and biasing^1,31^ also offer tunable nonlinearity, this typically comes with fixed sign (set by the attractive force) and order (set by device geometry).

Here, we experimentally demonstrate a nanomechanical resonator with an optically tunable, strong Duffing nonlinearity. Rather than from device geometry or material properties, this nonlinearity originates from cavity optomechanical backaction:^32^ it is linked to the intrinsic nonlinearity of the cavity optomechanical interaction and fully induced and controlled through laser light. Previously, light-controlled Duffing nonlinearity was observed in dissipatively coupled optomechanical systems^33,34^ for amplitudes of ∼100 nm and also predicted in coupled waveguides.^35^ We show that nanomechanical nonlinearity can generally emerge from a standard dispersively coupled driven cavity optomechanical resonator and can be orders of magnitude larger than intrinsic mechanical nonlinearity. In particular, the nonlinear shape of the cavity response as a function of displacement leads to a nonlinear optical spring effect, which results in tunable nonlinearities of different orders. We test this principle in an on-chip optomechanical device with exceptionally strong optomechanical coupling,^36^ so that motion explores a large fraction of the cavity response. We observe mechanical bistability for picometer-level vibrations, due to a Duffing nonlinearity whose sign and magnitude is completely controlled through an optical drive. Moreover, we demonstrate a scheme for realizing a pure intermode (cross-Kerr) nonlinearity between coupled resonators.

We consider a mechanical resonator with intrinsic resonance frequency Ω_m_ and mass *m*, whose displacement *x* linearly shifts the resonance frequency ω_*c*_ = ω_0_ – *Gx* of an optical cavity with optomechanical coupling strength *G*, nominal frequency ω_0_, and line width κ ≫Ω_m_ (Figure 1a).^32,37^ The latter condition—known as the fast-cavity or unresolved-sideband limit—implies that the cavity photon number *n*_c_ responds instantaneously on mechanical time scales, such that photon dynamics can be adiabatically eliminated. A spring laser with intensity *P*_in_ and frequency ω_L_, nominally detuned from the cavity by Δ_0_ = ω_L_ – ω_0_, drives it to a mean photon number *n*_c_. Under the nonlinear optomechanical interaction,^32^ the resonator displacement evolves as

1 $$mẍ=-kx-m\gamma ẋ+F_{\mathrm{opt}}$$

where $k=\Omega_{m}^{2}/m$ is the mechanical spring constant, γ its energy damping rate, and *F*_opt_ = *ℏGn*_c_ the radiation pressure force exerted by the intracavity field.

**Figure 1:**
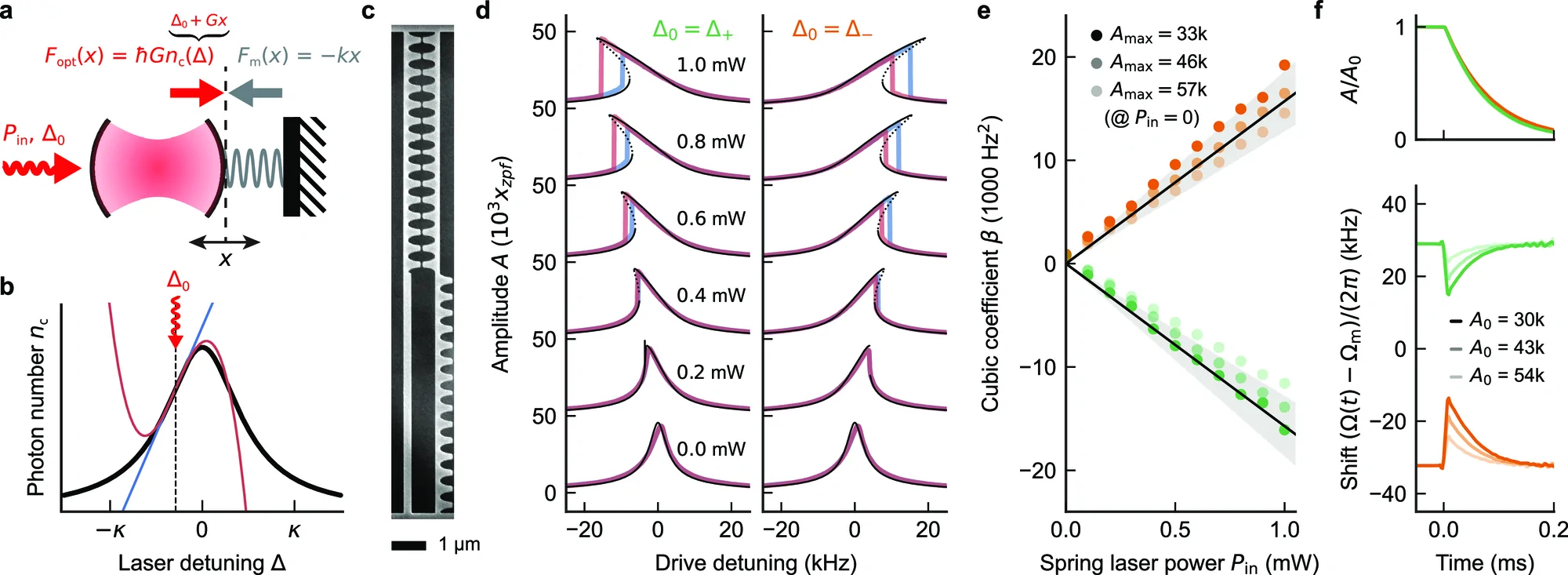
Laser-controlled Duffing nonlinearity. (a) Canonical optomechanical system: a cavity formed by two mirrors, where the movable right mirror experiences mechanical restoring force *F*_m_ and radiation pressure *F*_opt_. Mirror displacement *x* linearly shifts the detuning Δ between cavity and spring laser. We assume the fast-cavity limit κ ≫ Ω_m_, with κ the cavity line width and Ω_m_ the intrinsic mechanical frequency. (b) Cavity photon number *n*_c_ depends nonlinearly on Δ (black), resulting in optical backaction *F*_opt_ that is nonlinear in *x*. Tuning the nominal laser detuning Δ_0_ across the Lorentzian response controls this nonlinearity. At the indicated $\Delta_{0}=-\kappa /\left(2\sqrt{3}\right)$ , a linear approximation (blue) for small displacements *Gx* ≪ κ gives the optical spring effect; for larger displacements *Gx* ∼ κ, a cubic approximation (red) additionally yields Duffing nonlinearity. (c) Scanning electron micrograph of sliced nanobeam device. Flexural modes of the suspended silicon beams couple dispersively to a broadband photonic crystal cavity. (d) Driven response of the mechanical resonance at Ω_m_/2π = 17.6 MHz for positive detuning $\Delta_{+}=\kappa /\left(2\sqrt{3}\right)$ (left) and negative detuning $\Delta_{-}=-\kappa /\left(2\sqrt{3}\right)$ (right). Forward (blue) and backward (red) frequency sweeps are shown for increasing spring laser powers *P*_in_ (offset for clarity), with theory overlaid (black). (e) Cubic coefficients β for increasing *P*_in_ and drive amplitude *A*_max_. Estimates from the experimental response (circles) match well with eq 6 (black line) for both Δ_+_ (green, β < 0) and Δ_–_ (orange, β > 0). (f) Ring-down at *P*_in_ = 1.0 mW for Δ_+_ (green, shift >0 ) and Δ_–_ (orange, shift <0 ), for varying initial amplitude *A*_0_. The nonlinearity does not affect the resonator’s exponential amplitude decay (top), while it manifests in the evolving instantaneous frequency Ω­(*t*) once the mechanical drive is switched off for *t* > 0. Amplitudes are averaged over 1000 runs.

Importantly, *F*_opt_ depends on the displacement *x* as it shifts the instantaneous spring laser detuning Δ = Δ_0_ + *Gx*, which in turn modulates the intracavity photon number *n*_c_ = *n*_max_ *h*(2Δ/κ) through the Lorentzian cavity response

2 $$h\left(u\right)=\frac{1}{1+u^{2}}$$

Here, we define the maximum photon number *n*_max_ = 4*P*_in_/(*ℏω*_0_κ) attained on resonance (Δ = 0) and the relative detuning *u* = 2Δ/κ. For small displacements *Gx* ≪ κ, eq 2 can be linearized around the average relative detuning *u*_0_ = 2Δ_0_/κ, yielding an optical backaction force

3 $$F_{\mathrm{opt}}=F_{\mathrm{opt}}^{\mathrm{DC}}+\frac{2\hbar G^{2}n_{\max}h^{\prime }\left(u_{0}\right)}{\kappa }x=F_{\mathrm{opt}}^{\mathrm{DC}}-k_{\mathrm{opt}}x$$

that varies linearly with *x*. The static radiation pressure $F_{\mathrm{opt}}^{\mathrm{DC}}=\hbar Gn_{\max}h\left(u_{0}\right)$ only displaces the resonator’s equilibrium position *x*_0_, which we account for by redefining *x* → *x* – *x*_0_. Meanwhile, the optical spring constant *k*_opt_ supplements the intrinsic mechanical spring *k*, shifting the mechanical resonance frequency by

4 $$\delta \Omega =\frac{k_{\mathrm{opt}}}{2m\Omega_{m}}=-\frac{2g_{0}^{2}n_{\max}h^{\prime }\left(u_{0}\right)}{\kappa }$$

This well-known shift is conveniently expressed using the vacuum optomechanical coupling rate *g*_0_ = *Gx*_zpf_ and the resonator’s zero-point quantum fluctuation amplitude $x_{\mathrm{zpf}}=\sqrt{\hbar /\left(2m\Omega_{m}\right)}$ , even though eq 4 equally applies in the classical domain.

For larger amplitudes *Gx* ∼ κ, the instantaneous detuning samples a significant fraction of the cavity response, so that the nonlinear shape of *h*(*u*) needs to be taken into account. By moving the laser detuning *u*_0_ across this Lorentzian, we directly select and tune nonlinear terms in the optical force’s Taylor series—a distinct advantage over electrostatically induced nonlinearities.^1,31^ To induce a cubic nonlinearity to leading order, we select $u_{0}=u_{\pm }=\pm 1/\sqrt{3}$ (Figure 1b) where *h*″(*u*_0_) = 0. Consequently, |*h*′(*u*_0_)| is maximal, so that the linear spring shift |δΩ| is largest. The equation of motion

5 $$\mathrm{z̈}+\Omega^{2}z+\gamma ż+\beta z^{3}+O\left(z^{4}\right)=f\left(t\right)$$

of the resonator’s normalized displacement *z* = *x*/*x*_zpf_ is then the *Duffing equation*, featuring a cubic term with coefficient (see Supporting Information)­

6 $$\beta =-\frac{8\Omega_{m}n_{\max}h^{\prime \prime \prime }\left(u_{\pm }\right)g_{0}^{4}}{3\kappa^{3}}=-6\Omega_{m}\delta \Omega \frac{g_{0}^{2}}{\kappa^{2}}$$

In eq 5, Ω = Ω_m_ + δΩ is the spring-shifted linear frequency of the mechanical resonator and *f*(*t*) represents a driving force. We neglect the higher-order terms *z* ^*n*^ for *n* > 3 in the optical force as their coefficients scale with ${\left(g_{0}/\kappa \right)}^{n}\ll 1$ .

Crucially, this optomechanically induced Duffing coefficient β is fully and independently tunable in both magnitude and sign. By setting $u_{0}=-1/\sqrt{3}$ , the linear spring shift δΩ < 0 is negative, so that the corresponding β > 0 represents a hardening nonlinearity, while for $u_{0}=1/\sqrt{3}$ a softening nonlinearity β < 0 is obtained. Moreover, the magnitude of β scales linearly with δΩ and is, therefore, directly tuned by the spring laser power *P*_in_. This bidirectional control is qualitatively different from electrostatically tuned nonlinearities,^1,31^ where the effective Duffing coefficient generally combines a fixed intrinsic cubic term with a voltage-dependent electrostatic contribution. Because the electrostatic force scales as *V* ^2^, both its direct cubic contribution and the second-order correction it induces through the quadratic nonlinearity have definite sign, independent of the polarity of *V*. Electrostatic tuning can therefore only add softening: it can push an initially hardening resonator toward and into the softening regime, but never the reverse.

In principle, this optically tunable mechanical nonlinearity occurs in any fast-cavity optomechanical system. Whether it leads to observable effects depends on the vibrational amplitude. The critical phonon number *n*_NL_ above which a coherently driven resonator’s response becomes bistable^38^ occurs when the nonlinear mechanical frequency shift exceeds the line width γ. It can be expressed as

7 $$n_{\mathrm{NL}}=\frac{8}{3\sqrt{3}}\frac{\Omega_{m}\gamma }{\left|\beta \right|}=\frac{4}{9\sqrt{3}}\frac{\gamma }{\left|\delta \Omega \right|}\frac{\kappa^{2}}{g_{0}^{2}}$$

Owing to its large ratio *g*_0_/κ and fast-cavity operation, a well-suited optomechanical system to observe nonlinearity at low amplitude is the sliced nanobeam photonic crystal cavity,^39^ in which the effect of the nonlinear cavity response on the optical read-out of mechanical motion has been observed before^36^ as well as strong spring shifts.^40^ We employ this sliced nanobeam platform to demonstrate a controlled Duffing nonlinearity. Our on-chip device (Figure 1c) features a suspended silicon beam with a mechanical resonance at Ω_m_/2π = 17.6 MHz (effective mass *m* = 1 pg) coupled to a telecom optical mode (line width κ/2π = 313 GHz) at *g*_0_/2π = 3.5 ± 0.3 MHz. For this high-quality resonator (γ/2π = 4.1 kHz), we readily achieve spring shifts |δΩ| > 30 kHz when illuminating the cavity with a focused “spring” laser of up to ∼1 mW incident from free space. Here we remark that the cavity coupling efficiency is approximately 3%, so the coupled laser power is maximally a few tens of μW.

In Figure 1d, we show the resonator’s response to coherent driving *f*(*t*) = *f*_0_ cos­(ω_d_ *t*) at frequency ω_d_, for a range of incident spring laser powers *P*_in_ and detunings *u*_0_. The drive force *f*(*t*), with fixed amplitude *f*_0_, is applied optically by modulating an additional weak “force” laser resonant with the cavity, while a far-detuned “detect” laser reads out the resulting motion (see detailed method description in Supporting Information). When the spring laser is off, the resonator’s amplitude *A* follows a Lorentzian response that is approximately symmetric in frequency, indicating essentially linear resonator dynamics for this drive force strength, apart from a small residual shift arising from the detection laser and intrinsic nonlinearity (see below). Turning on the spring laser at detuning $\Delta_{0}=\kappa /\left(2\sqrt{3}\right)$ , where δΩ > 0, induces an observed softening nonlinearity that increases in strength with *P*_in_. The driving force is kept the same, so the optomechanically induced nonlinearity clearly exceeds the intrinsic mechanical nonlinearity of the beam. Notably, at higher *P*_in_ ≥ 0.6 mW, the resonator exhibits the bistable “shark fin” response typical for a Duffing resonator and associated hysteresis depending on the sweep direction of ω_d_. Conversely, for red-detuned $\Delta_{0}=-\kappa /\left(2\sqrt{3}\right)$ , where δΩ < 0, we find a hardening nonlinearity of similar strength but opposite sign. In that case, the characteristics of the response appear reflected in frequency. At both detunings, the experimental frequency response agrees well with Duffing theory (black curves in Figure 1d, see Supporting Information) for the coefficient β predicted by eq 6.

Notably, the minus sign in eq 6 implies that the nonlinear resonance shift, scaling as $∼\beta A^{2}/\left(\Omega_{m}x_{\mathrm{zpf}}^{2}\right)$ ,^38^ opposes the linear spring shift δΩ. This can be understood intuitively: at $u_{0}=\pm 1/\sqrt{3}$ the derivative |*h*′(*u*_0_)| is maximal, so that any detuning excursions will reduce the average derivative of the cavity response and, thus, the effective spring shift. Ultimately, for extremely large amplitudes *GA* ≫ κ, the cavity will be far off-resonant for most of the mechanical cycle, so that the resonator reverts back to its intrinsic resonance frequency (in the absence of other nonlinearities).

The cubic coefficient β can be extracted from the experimental data, by inverting the relation $A_{\max}=\sqrt{8\left(\omega_{\mathrm{d,max}}-\Omega \right)\Omega /\left(3\beta \right)}$ between the optimal drive frequency ω_d,max_ and amplitude *A*_max_ of the maximum response.^38^ As shown in Figure 1e, experimentally obtained estimates of β match well with eq 6, showcasing the full tunability of our scheme. At zero pump power, a small residual nonlinearity β < 900 Hz^2^ is estimated from the observed shift in the response peak. This is related to the intrinsic nonlinearity as well as the optomechanical nonlinearity of the detuned detection laser, which remains on even for zero spring laser power. We estimate the intrinsic geometrical nonlinearity contribution at β_intr_ ≈ 300 Hz^2^ from finite element modeling (Supporting Information) and the contribution from the detection laser at β_det_ ≈ −30 Hz^2^.

The largest observed optomechanical nonlinear coefficient β ≈ 16000 Hz^2^ is thus 50 times larger than the strength of the intrinsic nonlinearity. Appreciable Duffing nonlinearity emerges at critical amplitude $A_{\mathrm{NL}}=\sqrt{n_{\mathrm{NL}}}x_{\mathrm{zpf}}$ as low as *A*_NL_ = 17 000 *x*_zpf_, only 20 times larger than the thermal amplitude of our room-temperature resonator. Moreover, with *x*_zpf_ = 22 fm (estimated from simulations), *A*_NL_ = 0.36 nm represents a lateral displacement of less than a nanometer, much smaller than the critical amplitudes observed through different schemes^33^ or typical intrinsic mechanical nonlinearities for flexural nanomechanical resonators of similar dimensions.

We probe the time-domain dynamics of our nonlinear resonator in a ring-down experiment (Figure 1f). The resonator is first excited to a high amplitude *A*_0_ by a spring laser modulation, which is subsequently switched off at *t* = 0. Initially, for *t* < 0, the instantaneous frequency Ω­(*t*) of the resonator’s motion (see Supporting Information), shown in Figure 1f, bottom, is locked to the drive. After the modulation is switched off, the resonator is free to evolve at its natural frequency, which for a Duffing resonator depends on its amplitude.^3,5^ Consequently, at *t* = 0, the estimated instantaneous frequency Ω­(*t*) jumps to a nonlinearly shifted value. As the resonator’s amplitude rings down (top), this nonlinear shift gradually reduces, until the resonator oscillates at its linear (spring-shifted) frequency Ω.

We continue by exploring the resonator’s thermal dynamics in the presence of nonlinearity. Figure 2 shows the measured thermomechanical spectrum of the resonator while it is simultaneously driven by a force laser modulation at increasing depth *c*_d_. The coherent high-amplitude oscillations appear as a narrow peak with increasing amplitude, saturating the color scale. Notably, the broad spectrum of thermal vibrations now shifts in frequency, with the direction of the shift depending on the spring laser detuning: for $u_{0}=-1/\sqrt{3}$ it moves up in frequency as *c*_d_ increases, while for $u_{0}=1/\sqrt{3}$ it moves down. We note that additionally a weak, frequency-reflected band appears that moves in the other direction. This band predominantly reflects the resonator’s actual thermal motion, with only a small contribution from nonlinear mixing between thermal fluctuations and the strong coherent oscillation during transduction,^36^ and is related to thermal squeezing by the resonator’s nonlinear dynamics^11,41^ (see Supporting Information).

**Figure 2:**
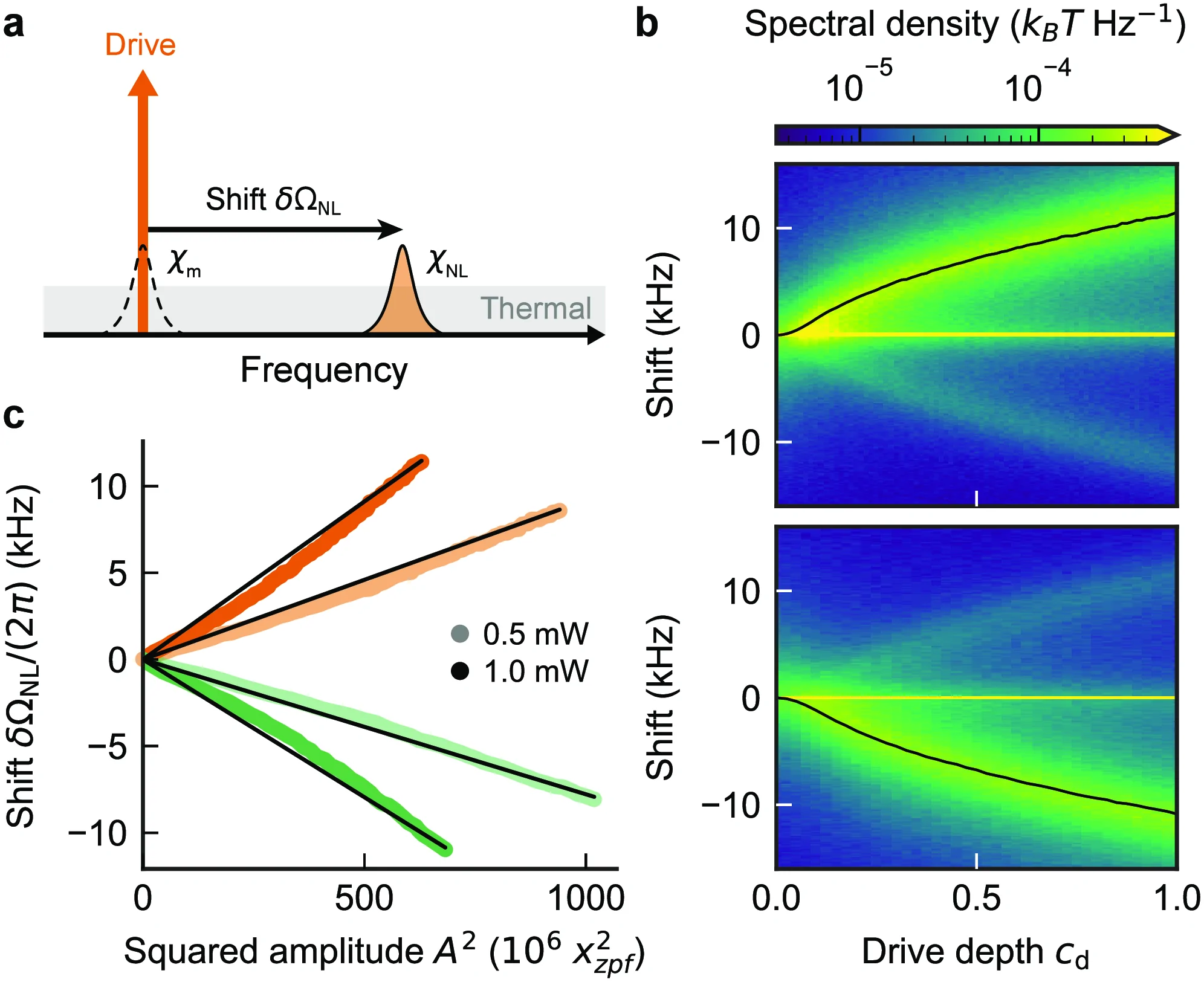
Nonlinear shift in susceptibility to thermal fluctuations. (a) A strong mechanical drive induces large amplitude oscillations, shifting the resonator’s susceptibility χ_NL_ to additional (thermal) forces. (b) Thermomechanical spectra of the resonator, coherently driven by modulations of a weak, resonant “force” laser at increasing depth *c*_d_. The strong (*P*_in_ = 1.0 mW) spring laser is detuned by $\Delta_{-}=-\kappa /\left(2\sqrt{3}\right)$ (top) or $\Delta_{+}=\kappa /\left(2\sqrt{3}\right)$ (bottom). The narrow line at zero detuning represents the resonator’s coherent response, while the two broader bands reflect the resonator’s thermal motion, with contributions from thermal squeezing^11,41^ and nonlinear transduction.^36^ (c) Fitted center frequencies of the Lorentzian thermal contribution plotted against the square of the driven amplitude, for Δ_–_ (orange) and Δ_+_ (green) at two powers *P*_in_. Expected susceptibility shifts from eq 8 (black) are overlaid in panels b and c.

Figure 2 demonstrates that in the presence of optomechanical nonlinearity, coherent oscillation alters the resonator’s susceptibility χ_NL_ to small thermal forces. Using harmonic balance^42^ (Supporting Information), we find that χ_NL_ remains Lorentzian like its linear counterpart χ_m_ (Figure 2a), but with a center frequency shifted by

8 $$\delta \Omega_{\mathrm{NL}}=\frac{3}{4}\frac{\beta a^{2}}{\Omega_{m}}=-\frac{9}{2}\delta \Omega \frac{g_{0}^{2}}{\kappa^{2}}a^{2}$$

where *a* = *A*/*x*_zpf_ is the normalized amplitude of the strong coherent oscillations. The thermal peak frequencies fitted from Figure 2b, plotted in Figure 2c against the measured coherent amplitude *A*, agree well with the predicted values from eq 8.

The above results demonstrate how nonlinear optical back-action can control the nonlinear dynamics of just a single mechanical mode. Interestingly, rich multimode nonlinear dynamics can be induced for multiple resonators that are simultaneously coupled to a common cavity. In that case, the cross-resonator optical spring, produced by their mutual back-action, can generate effective nonlinear hopping interactions between the mechanical modes. This has the prospect of creating cross-Duffing interactions, analogous to the cross-Kerr effect in optics and photonics. Such interactions are essential for a variety of fascinating phenomena, including nonlinear energy localization,^43^ frequency pulling in synchronization,^44^ correlated phases of matter,^45^ and nonlinear dynamical topological solitons.^28,29^

Here we analyze such coupled nonlinear dynamics. Two cavity-coupled resonators with displacements *x*_1_, *x*_2_ (Figure 3a) modulate the instantaneous cavity detuning Δ = Δ_0_ + *G*_1_ *x*_1_ + *G*_2_ *x*_2_ through their optomechanical coupling rates *G*_*j*_. Consequently, the optical force *F*_opt,*j*_(*x*_1_, *x*_2_) acting on each resonator *j* now depends on *both* their displacements. For small amplitudes, linearizing *F*_opt,*j*_(*x*_1_, *x*_2_) yields a cross-resonator optical spring that dynamically couples *x*_1_ ↔ *x*_2_ by a linear spring constant *k*_↔_ (see Supporting Information). However, for large displacements *G*_1_ *x*_1_, *G*_2_ *x*_2_ ∼ κ, the nonlinear cavity response must again be taken into account, resulting in a displacement-dependent cross-resonator spring *k*_↔_(*x*_1_, *x*_2_).

**Figure 3:**
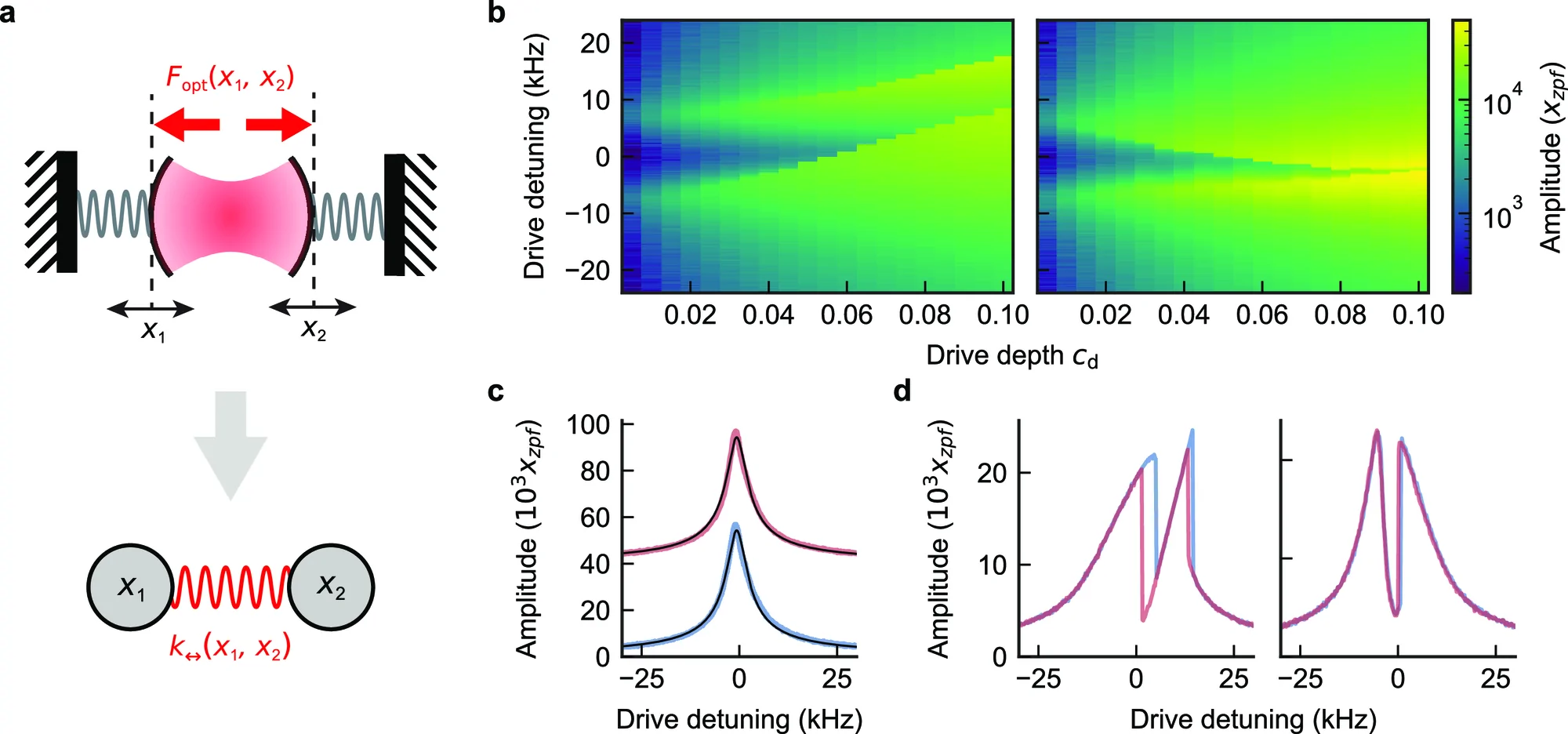
Optically mediated nonlinear coupling between mechanical resonators. (a) Multimode optomechanical system: both mirrors are free to move, so that photon number *n*_*c*_ depends nonlinearly on both displacements *x*_1_, *x*_2_ via the Lorentzian cavity response. The resulting radiation pressure *F*_opt_ on each mirror induces a nonlinear, amplitude-dependent optical spring *k*_↔_(*x*_1_, *x*_2_) coupling the two mechanical elements. (b, left) Forward-sweep frequency response of resonator 2 (Ω_m,2_/2π = 12.8 MHz) for increasing drive depth *c*_d_, while coupled to resonator 1 (Ω_m,1_/2π = 17.6 MHz) via a cross-resonator optical spring induced by modulating a single spring laser (detuning $\Delta_{-}=-\kappa /\left(2\sqrt{3}\right)$ ) at their difference frequency ΔΩ_12_. (b, right) Similar, but with an additional static spring laser at detuning $\Delta_{+}=\kappa /\left(2\sqrt{3}\right)$ that cancels the intraresonator nonlinearity while preserving the cross-resonator nonlinearity, resulting in reduced peak splitting with increasing amplitude. (c) Response of uncoupled resonator 1 with both spring lasers active. Cancellation of nonlinearity restores a Lorentzian line shape and eliminates hysteresis between forward (blue) and backward (red) sweeps (offset for clarity). (d) Response of coupled resonator 2 at fixed drive depth, for single-laser (left, *c*_d_ = 0.08) and two-laser (right, *c*_d_ = 0.045) driving. Hysteresis between forward (blue) and backward (red) sweeps is observed for single-laser driving, yet largely absent for two-laser driving.

The sliced photonic crystal nanobeam hosts several flexural mechanical overtones (Supporting Information) that couple to the cavity, allowing us to study such nonlinear coupling. We focus on a pair of modes that includes the aforementioned Ω_m,1_/2π = 17.6 MHz mode and a lower-frequency mode at Ω_m,2_/2π = 12.8 MHz with comparable damping rate γ_2_/2π = 3.7 kHz and vacuum optomechanical coupling rate *g*_0,2_/2π = 3.8 ± 0.1 MHz. With the spring laser, both modes experience spring shifts up to |δΩ_*j*_| ∼ 30 kHz so that they resonate at Ω_*j*_ = Ω_m,*j*_ + δΩ_*j*_. Due to the large frequency separation ΔΩ_12_ = Ω_1_ – Ω_2_ ≫ γ_*j*_, the static cross-resonator optical spring *k*_↔_ has virtually no effect on their dynamics. That frequency difference can, however, be overcome by modulating the intensity *P*_in_ of the spring laser at ΔΩ_12_. The resulting time-modulated spring *k*_↔_(*t*) stimulates frequency conversion between the modes by producing sidebands in the optical force that are mutually resonant. For small amplitudes, this leads to an effective linear hopping interaction that resonantly couples both modes at a rate $J=c_{h}\sqrt{\delta \Omega_{1}\delta \Omega_{2}}/2$ (Supporting Information), where *c*_h_ is the depth of the spring laser modulation.^40^

In Figure 3b (left panel), we probe the coherent response of the lower-frequency resonator 2 in the presence of a coupling modulation with depth *c*_h_ = 0.5, imprinted on the red-detuned $\left(u_{0}=-1/\sqrt{3}\right)$ spring laser. Here, resonator 2 is driven around its resonance frequency Ω_2_ by an additional weak modulation of the spring laser at depth *c*_d_. For small *c*_d_ < 0.02, two Lorentzian peaks are observed split by frequency 2*J*, signifying hybridization of the two mechanical resonators by the effective optical coupling. For stronger drives, the larger amplitude induces nonlinearity both in the individual optical springs *k*_opt,*j*_, as well as the cross-resonator spring *k*_↔_. The former increases the effective mechanical resonance frequencies at higher amplitudes, illustrated in Figure 3b, left, by the overall upshift in peak frequencies, while the latter reduces their effective splitting, which is proportional to the (amplitude-dependent) coupling rate. This combination of time-modulated drives and nonlinear optomechanical backaction was previously exploited to induce mode locking and synchronization.^9,10^

Conveniently, the cross-Duffing nonlinearity can be isolated by simultaneously applying *two* spring lasers at opposite detunings $u_{\pm }=\pm 1/\sqrt{3}$ to cancel the cubic nonlinearity of the individual resonators. As shown in Figure 3c, a symmetric mechanical response is then restored that exhibits no hysteresis, even at high drive power. By again modulating only one of the spring lasers at ΔΩ_12_, the nonlinearity in the cross-resonator spring *k*_↔_ is, nevertheless, induced (Supporting Information). As presented in Figure 3b (right panel), the response of resonator 2 then remains centered around Ω_2_ with increasing *c*_d_. For larger amplitudes, we do, however, see a strong reduction in the frequency splitting between the peaks, indicating a reduction in *k*_↔_ by the nonlinearity. This is also reflected in the example drive frequency response curves shown in Figure 3d: For a single modulated laser, these resemble two shark-fin-like curves for the two hybridized normal modes of the pair. With an isolated cross-Duffing nonlinearity, the response spectrum looks more symmetric, characterized by two peaks that tilt toward each other; shifting closer together as the amplitude increases.

Nonlinear coupling also impacts the resonator pair’s temporal dynamics. In the ringdown experiments shown in Figure 4a for a single spring laser at $u_{0}=-1/\sqrt{3}$ , resonator 1 is resonantly driven to amplitude *A*_0_ until *t* = 0, when the coupling tone at ΔΩ_12_ is switched on. For small amplitudes, we observe Rabi oscillations in which energy is exchanged between the resonators at the Rabi frequency *J* during ringdown. For larger amplitudes, the Rabi period increases—indicating a nonlinear reduction in the effective coupling rate *J*_NL_—while visibility reduces. The latter results from unequal intraresonator nonlinearities, which detunes their effective frequency difference from the coupling tone at ΔΩ_12_ causing incomplete Rabi oscillations.

**Figure 4:**
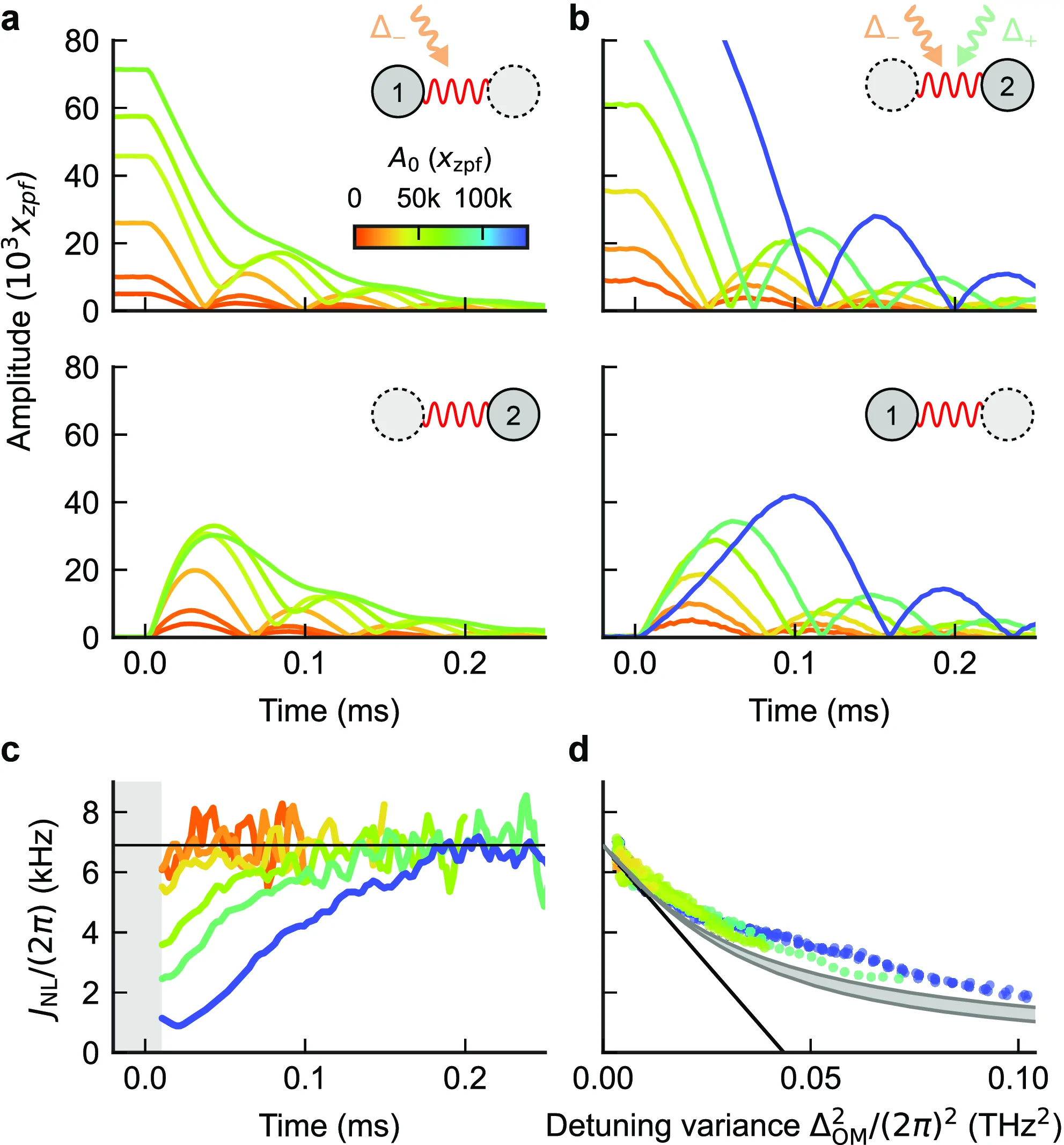
Time-evolution of nonlinearly coupled resonators. (a) Ring-down of coupled resonators 1 (top) and 2 (bottom) under single-laser driving at detuning $\Delta_{-}=-\kappa /\left(2\sqrt{3}\right)$ . For *t* < 0, resonator 1 is coherently excited to initial amplitude *A*_0_ (indicated by color) by modulating the spring laser. At *t* = 0, the drive tone is switched off and a coupling tone at the difference frequency ΔΩ_12_ is switched on, inducing Rabi oscillations whose period increases and visibility reduces at larger amplitudes. Amplitudes are averaged over 1000 runs. (b) Similar, but with an additional static spring laser at detuning $\Delta_{+}=\kappa /\left(2\sqrt{3}\right)$ and amplitude initialized in resonator 2. Cancellation of intraresonator nonlinearity keeps the coupling tone resonant to restore Rabi oscillation visibility, while the retained cross-resonator nonlinearity isolates the amplitude-dependent period lengthening. (c) Instantaneous coupling rates *J*_NL_(*t*) extracted from the coupled ring-downs in panel b. For small initial amplitudes *A*_0_, estimated *J*_NL_(*t*) approach the linear coupling *J*/2π ≈ 7 kHz (black) throughout. For larger drives, *J*_NL_ is initially reduced, recovering toward *J* as the amplitude dissipates. (d) Plotting the estimated *J*_NL_ against the detuning variance induced by both resonators reveals a clear trend independent of initial drive amplitude, with good agreement with eq 9 (black) for small to moderate mechanical amplitudes, while *J*_NL_ agrees well with numerical simulations at all amplitudes (gray, see Supporting Information).

With two spring lasers at *u*_±_ (Figure 4b), a pure cross-resonator nonlinearity is isolated. The coupling tone then remains resonant, so that full fringe visibility is recovered at high amplitudes. The effective Rabi rate *J*_NL_ clearly increases as the resonator amplitudes ring down. Figure 4c quantifies this behavior by extracting the instantaneous value of *J*_NL_ from the phase and amplitude of both resonators (Supporting Information). We see that *J*_NL_ starts out at a reduced effective coupling rate at *t* = 0 and then gradually increases to *J*/2π ≈ 7 kHz as the overall amplitude dissipates. Finally, Figure 4d plots the extracted values of *J*_NL_ against the summed squared amplitude of both resonators, expressed as the variance in the optomechanical detuning shift $\Delta_{\mathrm{OM}}^{2}=g_{0,1}^{2}a_{1}^{2}+g_{0,2}^{2}a_{2}^{2}$ , where *a*_*j*_ = *A*_*j*_/*x*_zpf,*j*_ is the normalized amplitude. We see a clear trend independent of the initial amplitude *A*_0_, which, for small $\Delta_{\mathrm{OM}}^{2}$ agrees well with the theoretically predicted

9 $$J_{\mathrm{NL}}=J\left[1-\frac{9}{4}\left(\frac{g_{0,1}^{2}}{\kappa^{2}}a_{1}^{2}+\frac{g_{0,2}^{2}}{\kappa^{2}}a_{2}^{2}\right)\right]$$

that we derive in the Supporting Information. Moreover, the extracted values of *J*_NL_ agree well with numerical simulations at *all* amplitudes (Supporting Information). These results illustrate the capability of optomechanical backaction to precisely control nonlinear coupling rates in multimode mechanical systems.

In conclusion, we have experimentally demonstrated that cavity optomechanical backaction can serve as a powerful and highly tunable source of nanomechanical nonlinearity. In the fast-cavity regime, the nonlinear Lorentzian response of the optical cavity produces a nonlinear optical spring that gives rise to strong Duffing dynamics. Using a sliced photonic crystal nanobeam cavity, we used this mechanism to realize optically induced nonlinearities that exceed the intrinsic mechanical nonlinearity by more than an order of magnitude, yielding subnanometer level critical nonlinear amplitudes. We note that the mechanism is very general and will occur in *any* cavity optomechanical resonator in the fast-cavity regime with standard dispersive optomechanical coupling. Even the current sliced nanobeam platform could be improved to yield still much stronger nonlinearity, as *g*_0_ values have been observed that exceed that in the current demonstration by a factor 10, and optical line widths κ that are at least 1 order of magnitude smaller.^36^ As the Duffing coefficient β scales with $g_{0}^{4}/\kappa^{3}$ , it is expected to be many orders of magnitude larger for optimized parameters. We do note that that increases higher-order nonlinearities by an even greater amount, resulting in an interesting trade-off depending on the specific target application.

For given device parameters, though, the maximum achievable nonlinearity is limited at extreme powers by two instabilities: for strong red-detuned driving *u*_0_ < 0, the negative linear optical spring softens the resonators until it cancels the intrinsic restoring force, while for strong blue-detuned driving *u*_0_ > 0, dynamical backaction introduces negative damping that eventually exceeds the intrinsic dissipation. As discussed in the Supporting Information, the maximum hardening nonlinearity at the optimal red-detuning $u_{0}=-1/\sqrt{3}$ is given by $\beta <3\Omega_{m}^{2}g_{0}^{2}/\kappa^{2}$ , while the softening (β < 0) nonlinearity at blue-detuned $u_{0}=1/\sqrt{3}$ is limited by $\beta >-g_{0}^{2}\gamma /\kappa$ .

A key feature of this nonlinearity is that it is fully controllable through the power and detuning of the external laser drives. We showed that the sign and magnitude of both self-Duffing and cross-Duffing interactions can be individually tuned by superposing multiple laser fields. Moreover, specific interactions can be addressed in the synthetic mode dimension through a judicious temporal modulation. This points to the possibility of engineering arbitrary nonlinear Hamiltonians in networks of resonators through spectral control of drive fields at frequency scales of the order of optical line widths and mechanical frequencies. Interactions can be dynamically reconfigured in situ fully optically without requiring changes to device geometry or material composition. Indeed, while we have focused here on the third-order Duffing nonlinearity, suitable laser drives could generate and optimize different nonlinearity orders related to the spectral shape of the optical forces. This represents a marked advantage over electrostatically tuned nonlinearities, which are governed by a single voltage and, therefore, lack independent control over the individual nonlinear coefficients.

Such fine-grained optical control could be exploited in a variety of contexts. At the level of individual resonators, optically induced Duffing nonlinearities may enable low-power bifurcation devices, nonlinear sensing schemes, mechanical logic elements, and tunable susceptibilities for noise manipulation and squeezing. In multimode systems, engineered nonlinear couplings provide a promising platform for exploring collective nonlinear dynamics, synchronization, nonlinear transport, and driven-dissipative phases in coupled oscillator networks.^19–21^ In particular, the realization of controllable cross-Duffing interactions could enable nonlinear topological phenomena and soliton formation in optomechanical lattices.^28,29^ In the quantum regime, nonlinear backaction provides a powerful approach to the engineering of nonclassical quantum states and mechanical qubits.^46,47^

More broadly, these results establish cavity optomechanics as a versatile framework for realizing reconfigurable nonlinear dynamics on the nanoscale. Combined with the scalability of nano-optomechanical architectures and the possibility of measurement and control down to the quantum regime, this provides a powerful setting for exploiting controlled nonlinearity for fundamental studies as well as technological applications of nonlinear resonators and networks.

## Supplementary Material



## Notes

Detailed derivations of the main results, finite element estimation of the intrinsic nonlinearity, experimental methods, details on data analysis, and numerical simulations of coupled resonators (PDF)

## References

[1] Lifshitz, R. ; Cross, M. C. In Reviews of nonlinear dynamics and complexity; Wiley-VCH Verlag GmbH & Co. KGaA, 2008; pp 1–52.

[2] Guckenheimer, J. ; Holmes, P. Nonlinear oscillations, dynamical Systems, and bifurcations of vector fields; Applied Mathematical Sciences, Vol. 42; Springer: New York, NY, 1983.

[3] Jordan, D. W. ; Smith, P. Nonlinear ordinary differential equations: An introduction for scientists and engineers, 4th ed; Oxford University Press: Oxford, 2007.

[4] Holmes P., Rand D. (1976). The bifurcations of Duffing’s
Equation: An application of
catastrophe theory. J. Sound Vibr..

[5] Kovacic, I. ; Brennan, M. J. The Duffing equation: Nonlinear oscillators and their phenomena; Wiley: Chichester, West Sussex, U.K., 2011.

[6] Mahboob I., Flurin E., Nishiguchi K., Fujiwara A., Yamaguchi H. (2011). Interconnect-free
parallel logic circuits in a single mechanical resonator. Nat. Commun..

[7] Mahboob I., Okamoto H., Yamaguchi H. (2016). An electromechanical
Ising Hamiltonian. Sci. Adv..

[8] Romero E., Mauranyapin N. P., Hirsch T. M., Kalra R., Baker C. G., Harris G. I., Bowen W. P. (2024). Acoustically driven single-frequency
mechanical logic. Phys. Rev. Applied.

[9] Mercadé L., Pelka K., Burgwal R., Xuereb A., Martínez A., Verhagen E. (2021). Floquet Phonon Lasing
in Multimode Optomechanical Systems. Phys. Rev.
Lett..

[10] Asano M., Okamoto H., Yamaguchi H. (2025). Synthesized
Kuramoto Potential via
Optomechanical Floquet Engineering. Science
Advances.

[11] Huber J. S., Rastelli G., Seitner M. J., Kölbl J., Belzig W., Dykman M. I., Weig E. M. (2020). Spectral evidence
of squeezing of a weakly damped driven nanomechanical mode. Phys. Rev. X.

[12] Serra-Garcia M., Foehr A., Molerón M., Lydon J., Chong C., Daraio C. (2016). Mechanical autonomous stochastic heat engine. Phys. Rev. Lett..

[13] Kippenberg T. J., Gaeta A. L., Lipson M., Gorodetsky M. L. (2018). Dissipative
Kerr solitons in optical microresonators. Science.

[14] Ochs J. S., Boneß D. K. J., Rastelli G., Seitner M., Belzig W., Dykman M. I., Weig E. M. (2022). Frequency comb from a single driven
nonlinear nanomechanical mode. Phys. Rev. X.

[15] Sun J., Yang W., Zheng T., Xiong X., Liu Y., Wang Z., Li Z., Zou X. (2021). Novel nondelay-based
reservoir computing with a single micromechanical nonlinear resonator
for high-efficiency information processing. Microsyst. & Nanoeng..

[16] Tanaka G., Yamane T., Héroux J. B., Nakane R., Kanazawa N., Takeda S., Numata H., Nakano D., Hirose A. (2019). Recent advances
in physical reservoir computing: A review. Neural
Networks.

[17] Marković D., Mizrahi A., Querlioz D., Grollier J. (2020). Physics for neuromorphic
computing. Nat. Rev. Phys..

[18] Hartmann M. J., Brandão F. G. S. L., Plenio M. B. (2006). Strongly interacting
polaritons in coupled arrays of cavities. Nat.
Phys..

[19] Ludwig M., Marquardt F. (2013). Quantum many-body
dynamics in optomechanical arrays. Phys. Rev.
Lett..

[20] Leykam D., Chong Y. D. (2016). Edge solitons in
nonlinear-photonic topological insulators. Phys.
Rev. Lett..

[21] Meier E. J., An F. A., Gadway B. (2016). Observation
of the topological soliton
state in the Su–Schrieffer–Heeger model. Nat. Commun..

[22] Mukherjee S., Rechtsman M. C. (2020). Observation
of Floquet solitons in a topological bandgap. Science.

[23] Mukherjee S., Rechtsman M. C. (2021). Observation of unidirectional solitonlike edge states
in nonlinear Floquet topological insulators. Phys. Rev. X.

[24] Lin S., Zhang L., Tian T., Duan C.-K., Du J. (2021). Dynamic observation
of topological soliton states in a programmable nanomechanical lattice. Nano Lett..

[25] Kirsch M. S., Zhang Y., Kremer M., Maczewsky L. J., Ivanov S. K., Kartashov Y. V., Torner L., Bauer D., Szameit A., Heinrich M. (2021). Nonlinear second-order photonic topological
insulators. Nat. Phys..

[26] Jürgensen M., Mukherjee S., Rechtsman M. C. (2021). Quantized nonlinear Thouless pumping. Nature.

[27] Jürgensen M., Mukherjee S., Jörg C., Rechtsman M. C. (2023). Quantized
fractional Thouless pumping of solitons. Nat.
Phys..

[28] Hadad Y., Khanikaev A. B., Alù A. (2016). Self-induced topological transitions
and edge states supported by nonlinear staggered potentials. Phys. Rev. B.

[29] Hadad Y., Vitelli V., Alu A. (2017). Solitons and propagating domain walls
in topological resonator arrays. ACS Photon.

[30] Hadad Y., Soric J. C., Khanikaev A. B., Alù A. (2018). Self-induced
topological protection in nonlinear circuit arrays. Nat. Electron..

[31] Kozinsky I., Postma H. W. C., Bargatin I., Roukes M. L. (2006). Tuning
Nonlinearity,
Dynamic Range, and Frequency of Nanomechanical Resonators. Appl. Phys. Lett..

[32] Aspelmeyer M., Kippenberg T. J., Marquardt F. (2014). Cavity optomechanics. Rev. Mod.
Phys..

[33] Li H., Chen Y., Noh J., Tadesse S., Li M. (2012). Multichannel
cavity optomechanics for all-optical amplification of radio frequency
signals. Nat. Commun..

[34] Westwood-Bachman J. N., Firdous T., Kobryn A. E., Belov M., Hiebert W. K. (2022). Isolation
of optomechanical nonlinearities in nanomechanical cantilevers. Phys. Rev. A.

[35] Ashour M., Caspers J. N., Weig E. M., Degenfeld-Schonburg P. (2021). Spontaneous
parametric down-conversion induced by optomechanical gradient forces
in nanophotonic waveguides. Phys. Rev. A.

[36] Leijssen R., La Gala G. R., Freisem L., Muhonen J. T., Verhagen E. (2017). Nonlinear
cavity optomechanics with nanomechanical thermal fluctuations. Nat. Commun..

[37] Bowen, W. P. ; Milburn, G. J. Quantum Optomechanics; CRC Press, Taylor & Francis Group, 2016.

[38] Schmid, S. ; Villanueva, L. G. ; Roukes, M. L. Fundamentals of nanomechanical resonators; Springer International Publishing: Cham, 2023.

[39] Leijssen R., Verhagen E. (2015). Strong optomechanical interactions
in a sliced photonic
crystal nanobeam. Sci. Rep..

[40] Mathew J. P., del Pino J., Verhagen E. (2020). Synthetic gauge fields for phonon
transport in a nano-optomechanical system. Nat.
Nanotechnol..

[41] Fani
Sani F., Rodrigues I. C., Bothner D., Steele G. A. (2021). Level attraction
and idler resonance in a strongly driven Josephson cavity. Phys. Rev. Research.

[42] Krack, M. ; Gross, J. Harmonic balance for nonlinear vibration problems; Mathematical Engineering; Springer International Publishing: Cham, 2019.

[43] Sato M., Hubbard B. E., Sievers A. J. (2006). Colloquium:
Nonlinear energy localization
and its manipulation in micromechanical oscillator arrays. Rev. Mod. Phys..

[44] Matheny M. H., Grau M., Villanueva L. G., Karabalin R. B., Cross M. C., Roukes M. L. (2014). Phase synchronization
of two anharmonic
nanomechanical oscillators. Phys. Rev. Lett..

[45] Jin J., Rossini D., Fazio R., Leib M., Hartmann M. J. (2013). Photon
solid phases in driven arrays of nonlinearly coupled cavities. Phys. Rev. Lett..

[46] Clarke, J. ; Neveu, P. ; Verhagen, E. ; Vanner, M. R. Deterministic Mechanical Wigner Negativity via Nonlinear Cavity Quantum Optomechanics in the Unresolved-Sideband Regime; arXiv:quant-ph, 2025, 10.48550/arXiv.2505.01942.

[47] Samanta C., Bonis S. L. D., Møller C. B., Tormo-Queralt R., Yang W., Urgell C., Stamenic B., Thibeault B., Jin Y., Czaplewski D. A., Pistolesi F., Bachtold A. (2023). Nonlinear nanomechanical resonators approaching the
quantum ground state. Nat. Phys..

