# Supplementary material for: Strong Nanomechanical Duffing Nonlinearity and Interactions Induced through Cavity Optomechanics
Source: Nano Lett. 2026 Sep 8;26(37):12334–41. doi: 10.1021/acs.nanolett.6c02784 (PMC13614301; doi:10.1021/acs.nanolett.6c02784)
Supplement: Supplementary file 1 [file nl6c02784_si_001.pdf]

Supplementary Information for: "Strong nanomechanical Duffing nonlinearity and interactions induced through cavity optomechanics"

Jesse J. Slim[1,2] and Ewold Verhagen[1]

[1] *Center for Nanophotonics, AMOLF, Science Park 104, 1098 XG Amsterdam, The Netherlands*
[2] *Australian Research Council Centre of Excellence in Quantum Biotechnology (QUBIC), School of Mathematics and Physics, University of Queensland, St Lucia, Queensland 4072, Australia*

CONTENTS

I. Nonlinear optical backaction 1
  A. Limits on achievable nonlinearity 2

II. Frequency response of a Duffing resonator 2

III. Thermal sidebands in nonlinear resonators 3

IV. Effective nonlinear interactions 4

V. Simulating geometric nonlinearity 6

VI. Experimental methods 6
  A. Optomechanical system 6
  B. Excitation and readout 6

VII. Extracting instantaneous oscillation frequencies 7

VIII. Estimating instantaneous coupling rates 7
  A. Correcting for local oscillator detuning 7
  B. Relative amplitude correction 8

IX. Simulating coupled resonators with full Lorentzian cavity response 8

References 9

I. NONLINEAR OPTICAL BACKACTION

In this section, we present a detailed analysis of optical backaction in fast-cavity optomechanical systems. We assume operation in the fast-cavity (unresolved sideband) regime [1]. We start from the equation of motion

$$m\ddot{x} = -kx - m\gamma\dot{x} + F_{\mathrm{opt}}, \tag{S1}$$

for a mechanical resonator subject to radiation pressure force F_{opt}, given in the main text as Eq. (1). Optical backaction arises as the optical force

$$F_{\mathrm{opt}} = \hbar G n_{\mathrm{c}} = \hbar G n_{\mathrm{max}} h\left(\frac{2\Delta_0}{\kappa} + \frac{2Gx}{\kappa}\right) \tag{S2}$$

depends instantaneously on the mechanical displacement x, through the optomechanical coupling G and the Lorentzian cavity response $h(u)$. This treatment is valid in the fast-cavity limit where the cavity linewidth $\kappa \gg \Omega_{\mathrm{m}}$ is much larger than the mechanical frequency, so that the photon dynamics can be eliminated adiabatically.

To analyse the nonlinear relation between F_{opt} and x, we expand $h(u)$ into its Taylor series around $u_0 = 2\Delta_0/\kappa$ and obtain

$$F_{\mathrm{opt}} = \hbar G n_{\mathrm{max}}\Bigg[h(u_0) + h'(u_0)\left(\frac{2Gx}{\kappa}\right) + \frac{h''(u_0)}{2}\left(\frac{2Gx}{\kappa}\right)^2 + \frac{h'''(u_0)}{6}\left(\frac{2Gx}{\kappa}\right)^3 + \mathcal{O}(x^4)\Bigg]. \tag{S3}$$

The constant term $F_{\mathrm{opt}}^{\mathrm{DC}} = \hbar G n_{\mathrm{max}} h(u_0)$ represents static radiation pressure, which displaces the resonator's equilibrium position to x_0. Conventionally, this is accounted for by redefining $x \to x - x_0$.

The linear term

$$F_{\mathrm{opt}}^{(1)} = \frac{2\hbar n_{\mathrm{max}} h'(u_0) G^2}{\kappa} x = -k_{\mathrm{opt}} x \tag{S4}$$

gives rise to the conventional, linear optical spring effect, characterised by spring constant k_{opt} that supplements the intrinsic mechanical spring constant $k = m\Omega_{\mathrm{m}}^2$. It shifts the mechanical frequency by

$$\delta\Omega = \Omega - \Omega_{\mathrm{m}} = \sqrt{\frac{k + k_{\mathrm{opt}}}{m}} - \sqrt{\frac{k}{m}} \tag{S5}$$

$$= \Omega_{\mathrm{m}}\left(\sqrt{1 + k_{\mathrm{opt}}/k} - 1\right) \tag{S6}$$

$$\approx \frac{\Omega_{\mathrm{m}} k_{\mathrm{opt}}}{2k} = \frac{k_{\mathrm{opt}}}{2m\Omega_{\mathrm{m}}}, \tag{S7}$$

where the approximation is valid for small optical spring $k_{\mathrm{opt}} \ll k$. Plugging in k_{opt} yields

$$\delta\Omega = -\frac{\hbar n_{\mathrm{max}} h'(u_0) G^2}{m\Omega_{\mathrm{m}}\kappa} = -\frac{2 n_{\mathrm{max}} h'(u_0) g_0^2}{\kappa}, \tag{S8}$$

where we have substituted the vacuum optomechanical coupling rate $g_0 = G x_{\mathrm{zpf}}$ and zero-point fluctuation amplitude $x_{\mathrm{zpf}} = \sqrt{\hbar/(2m\Omega_{\mathrm{m}})}$. This relation for the linear optical spring shift is given in the main text as Eq. (4).

Next, we express the mechanical equation of motion using the normalized displacement $z = x/x_{\mathrm{zpf}}$,

$$\ddot{z} = -\Omega_{\mathrm{m}}^2 z - \gamma\dot{z} + f_{\mathrm{opt}}(z), \tag{S9}$$

where $f_{\mathrm{opt}}(z) = F_{\mathrm{opt}}/(m x_{\mathrm{zpf}})$ is the normalized optical force. For reference, we give the linear term in the normalized force as

$$f_{\mathrm{opt}}^{(1)} = \frac{4\Omega_{\mathrm{m}} n_{\mathrm{max}} h'(u_0) g_0^2}{\kappa} z = -2\Omega_{\mathrm{m}}\delta\Omega\, z \tag{S10}$$

The quadratic term in Eq. (S3),

$$F_{\rm opt}^{(2)} = \frac{2\hbar G^3 n_{\rm max} h''(u_0)}{\kappa^2} x^2, \tag{S11}$$

yields a contribution

$$f_{\rm opt}^{(2)} = \frac{4\Omega_{\rm m} n_{\rm max} h''(u_0) g_0^3}{\kappa^2} z^2 = -\alpha z^2 \tag{S12}$$

to the normalized optical force. However, precisely at the spring laser detunings $u_0 = u_\pm = \pm 1/\sqrt{3}$ that we use, where the optical spring shift $\delta\Omega$ is maximally positive or negative, respectively, the second derivative $h''(u_\pm)$ vanishes. Consequently, the quadratic nonlinear coefficient α induced by the spring laser is zero in all of our experiments.

In contrast, the cubic term in Eq. (S3),

$$F_{\rm opt}^{(3)} = \frac{8\hbar n_{\rm max} h'''(u_0) G^4}{6\kappa^3} x^3, \tag{S13}$$

does not vanish at $u_0 = u_\pm$. When normalized, the cubic optical force term reads

$$f_{\rm opt}^{(3)} = \frac{8\Omega_{\rm m} n_{\rm max} h'''(u_0) g_0^4}{3\kappa^3} z^3 = -\beta z^3, \tag{S14}$$

where β is the cubic nonlinear coefficient. This coefficient can be conveniently written using the linear spring shift $\delta\Omega$ as

$$\beta = \Omega_{\rm m} \delta\Omega \frac{4h'''(u_0)}{3h'(u_0)} \frac{g_0^2}{\kappa^2}. \tag{S15}$$

This expression is valid for any spring laser detuning u_0. At $u_0 = u_\pm$, we find $h'''(u_\pm)/h'(u_\pm) = -9/2$, so that we can write

$$\beta = -6\Omega_{\rm m} \delta\Omega \frac{g_0^2}{\kappa^2}, \tag{S16}$$

given in the main text as Eq. (6).

A. Limits on achievable nonlinearity

For pure radiation-pressure coupling between the mechanical and optical resonators (i.e., in the absence of optothermal interactions [2]), the maximum achievable nonlinearity is limited by two instabilities at extreme powers: for strong red-detuned driving $u_0 < 0$, the negative linear optical spring softens the resonators until it cancels the intrinsic restoring force, while for strong blue-detuned driving $u_0 > 0$, dynamical backaction introduces negative damping that eventually exceeds the intrinsic dissipation. We explore both limits in the following.

For red-detuned drives, we require that the optical spring constant, given by Eq. (S4), is less than the intrinsic spring,

$$m\Omega_{\rm m}^2 = k > k_{\rm opt} = \frac{2\hbar n_{\rm max} h'(u_0) G^2}{\kappa}. \tag{S17}$$

This is conveniently rewritten as a limit on the drive power

$$g_0^2 n_{\rm max} < \frac{\Omega_{\rm m}\kappa}{4h'(u_0)}. \tag{S18}$$

Plugging this into Eq. (S14) for the cubic coefficient β yields a limit on the maximum hardening nonlinearity

$$\beta = -\frac{8\Omega_{\rm m} n_{\rm max} h'''(u_0) g_0^4}{3\kappa^3} < -\Omega_{\rm m}^2 \frac{2h'''(u_0)}{3h'(u_0)} \frac{g_0^2}{\kappa^2}. \tag{S19}$$

At the optimal red-detuning $u_0 = -1/\sqrt{3}$, this limit reduces to

$$\beta < 3\Omega_{\rm m}^2 \frac{g_0^2}{\kappa^2}. \tag{S20}$$

This limitation does not occur in the same way for blue detuning. There, however, the change of dissipation affects the (nonlinear) dynamics through reducing the mechanical linewidth. Importantly, we note that in the fast-cavity limit (unresolved sideband regime), dissipation rate changes [1]

$$\gamma_{\rm opt} = \frac{-32 g_0^2 n_{\rm max} \Omega_{\rm m}}{\kappa^2} \frac{u_0}{(1+u_0)^3} \tag{S21}$$

are suppressed (by a factor $\sim \Omega_{\rm m}/\kappa$) and much smaller than the optical spring shift. Nonetheless, parametric instability is reached for blue detuning when $\gamma_{\rm opt} > -\gamma$. From this condition, we derive a limit on the maximum power for a blue-detuned drive,

$$g_0^2 n_{\rm max} < \frac{\gamma\kappa^2}{32\Omega_{\rm m}} \frac{(1+u_0^2)^3}{u_0}. \tag{S22}$$

This yields a limit on the maximum softening ($\beta < 0$) nonlinearity

$$-\beta < \frac{g_0^2\gamma}{12\kappa} \frac{(1+u_0^2)^3 h'''(u_0)}{u_0}, \tag{S23}$$

which, at the optimal blue-detuning $u_0 = 1/\sqrt{3}$ reduces to

$$-\beta < \frac{g_0^2\gamma}{\kappa}. \tag{S24}$$

Finally, we note that, in both cases, the maximum nonlinearity can be improved by increasing the optomechanical coupling rate g_0 or reducing the cavity linewidth κ.

II. FREQUENCY RESPONSE OF A DUFFING RESONATOR

In this section, we analyse the frequency response of a Duffing resonator to a strong ‘pump’ force, optionally supplemented by a weak ‘probe’ force. We do so using the method of harmonic balance [3]. This approach,

commonly used to evaluate the dynamics of Duffing oscillators [4], relies on expanding the displacement $z(t)$ as a Fourier series and then truncating that expansion to a specified order, while balancing the amplitudes of the remaining harmonics.

We start from the equation of motion

$$\ddot{z} + \gamma\dot{z} + \Omega^2 z + \beta z^3 = f(t) \tag{S25}$$

that describes the evolution of a general Duffing oscillator subject to a two-tone driving force

$$f(t) = f_1 \cos(\omega_1 t + \psi_1) + f_2 \cos(\omega_2 t + \psi_2) \tag{S26}$$

that includes a strong 'pump' tone with amplitude f_1 and frequency ω_1 and a weak 'probe' tone with amplitude f_2 and frequency ω_2. For simplicity, we assume that the frequencies ω_1, ω_2 are incommensurate, such that the phase offsets are not essential and we can set $\phi_1 = \phi_2 = 0$ without affecting the dynamics.

In response to this composite force, we seek an approximate solution to the resonator evolution of the form

$$z(t) = a_1 \cos(\omega_1 t + \phi_1) + a_2 \cos(\omega_2 t + \phi_2). \tag{S27}$$

We insert Eq. (S27) into the Duffing equation (S25) and balance the fundamental harmonics at ω_1 and ω_2. First, we solve for the pump amplitude a_1 while neglecting the probe amplitude ($a_2 = 0$). This results in the well-known equation

$$a_1^2 \left[\left(\omega_1^2 - \Omega^2 - \frac{3}{4}\beta a_1^2 \right)^2 + (\gamma\omega_1)^2 \right] = f_1^2 \tag{S28}$$

that describes the approximate frequency response of a Duffing oscillator to harmonic driving. This equation underlies the theory curves plotted in Fig. 1d. For driving $f_1 < f_1^c$ below the critical driving amplitude, Eq. (S28) admits a single solution for the amplitude a_1, while for strong driving $f_1 > f_1^c$ the amplitude becomes multivalued and hysteresis emerges.

Next, we solve for the probe amplitude a_2 in the presence of the pump. After balancing the fundamental harmonics, we find

$$a_2^2 \left[\left(\omega_2^2 - \Omega^2 - \frac{3}{4}\beta(2a_1^2 + a_2^2) \right)^2 + (\gamma\omega_1)^2 \right] = f_2^2 \tag{S29}$$

Assuming that $a_1 \gg a_2$ in the term in brackets, we arrive at the approximation

$$a_2^2 = \frac{f_2^2}{(\mu^2 - \omega_2^2)^2 + \gamma^2\omega_2^2} \tag{S30}$$

for the squared amplitude of motion at the probe frequency. This corresponds exactly to the susceptibility of a linear harmonic oscillator with natural frequency μ. The shifted frequency μ satisfies the expression

$$\mu^2 = \Omega^2 + \frac{3}{2}\beta a_1^2. \tag{S31}$$

After plugging in Eq. (S16) for the radiation-pressure-induced β, we arrive at the expression

$$\delta\Omega_{\mathrm{NL}} = \mu - \Omega \approx \frac{3}{4}\frac{\beta a_1^2}{\Omega_{\mathrm{m}}} = -\frac{9}{2}\delta\Omega\frac{g_0^2}{\kappa^2}a_1^2 \tag{S32}$$

that describes this nonlinear contribution to the spring shift of the *probe* susceptibility, valid under the assumption $\delta\Omega_{\mathrm{NL}} \ll \Omega$. This equation is given in the main text as Eq. (8). Although Eq. (S32) is derived for a coherent probe tone, it equally predicts the nonlinearly shifted response to weak, incoherent thermal forces, as shown in Fig. 2.

III. THERMAL SIDEBANDS IN NONLINEAR RESONATORS

In this section, we discuss the appearance of two thermal sidebands in Fig. 2 and compare to the experiment presented in Ref. [5].

For the electromechanical resonator of Ref. [5], two thermal sidebands were observed when the resonator was driven to a coherent amplitude large enough to induce nonlinear dynamics. As detailed there, both sidebands reflect the resonator's actual thermal motion and arise from squeezing of thermal fluctuations by the resonator's nonlinear dynamics. In that experiment, nonlinearity enters only through the resonator's equation of motion, while the transduction of that motion remains linear over the relevant range. Our experiments, in contrast, additionally exhibit nonlinear mixing in readout [6], which generally introduces ambiguity about the origin of any additional sidebands.

We quantify the effect of nonlinear transduction. For a weak sinusoidal vibration at $\Omega_{\mathrm{m}} + \delta\Omega_{\mathrm{NL}}$, we calculate the ratio of its nonlinear transduction at $\Omega_{\mathrm{m}} - \delta\Omega_{\mathrm{NL}}$ to its linear transduction, as expected from third order mixing with the strong coherent pump at Ω_{m}. This ratio

$$\frac{\mathcal{I}_{\mathrm{NL}}}{\mathcal{I}_{\mathrm{L}}} = \frac{1}{8}\frac{\mathcal{R}'''(u_{\mathrm{det}})}{\mathcal{R}'(u_{\mathrm{det}})}\left(\frac{2g_0}{\kappa}\right)^2 a^2 \tag{S33}$$

depends on the first- and third-order derivatives of the (Fano-shaped) cavity reflectivity $\mathcal{R}(u)$ at the nondimensional 'detect' laser detuning $u_{\mathrm{det}} = 2\Delta_{\mathrm{det}}/\kappa \approx -5.0$, as well as the coherent pump's normalized amplitude $a = A/x_{\mathrm{zpf}}$, vacuum optomechanical coupling rate g_0 and cavity linewidth κ.

As shown in Fig. S1, the measured value of this ratio for the thermal sidebands shown in Fig. 2b, top, is at least an order of magnitude larger than would be expected from Eq. (S33). Moreover, it does not show quadratic scaling with the pump amplitude a. These observations indicate that nonlinear mixing alone cannot explain the appearance of frequency-reflected 'ghost' bands—they must, to a large part, reflect the resonator's actual motion.

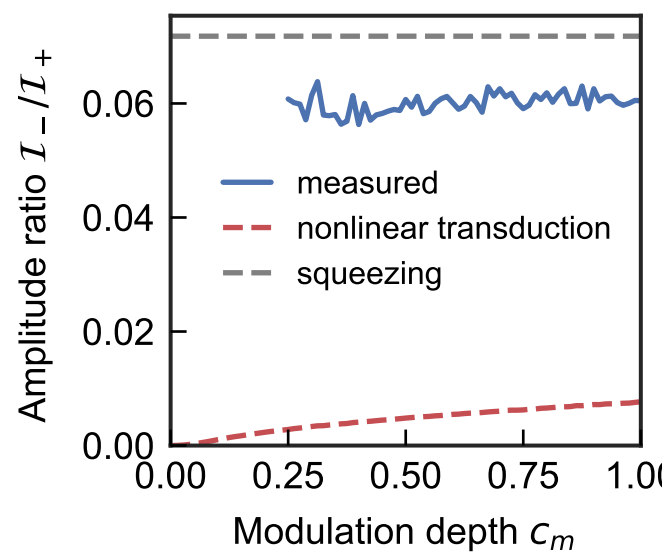

FIG. S1. Ratio of the amplitudes of the lower ($\mathcal{I}_-$) and upper ($\mathcal{I}_+$) thermal sidebands in Fig. 2b, top. Measured values (blue) are extracted by fitting two Lorentzians. Amplitude ratios expected from third-order nonlinear mixing between the strong coherent motion and the upper thermal sideband in transduction (red) are at least an order of magnitude smaller. The ratio expected from squeezing by nonlinear dynamics [5] (gray) is slightly larger.

Next, we calculate the sideband amplitude ratio related to the squeezing effect described in [5],

$$\frac{\mathcal{I}_-}{\mathcal{I}_+} = \tanh(\varphi_{\rm hi})^2, \tag{S34}$$

given here as the inverse of Eq. (12) in [5]. We expect to be on the 'high' amplitude branch, so that $\varphi_{\rm hi}$ is the squeezing parameter as defined in Eq. (10) of [5]. For resonant driving, the squeezing parameter satisfies $\exp(4\varphi_{\rm hi}) = 3$, independent of the coherent amplitude a or the nonlinear coefficient, so that the ratio $\mathcal{I}_-/\mathcal{I}_+ \approx 0.072$ is fixed. As shown in Fig. S1, this matches our measured values to within 15%. The remaining discrepancy is perhaps related to our resonator's $\sim 100\times$ lower quality factor compared to the experiment in [5], as the analysis in [5] neglects certain contributions scaling with γ^2.

IV. EFFECTIVE NONLINEAR INTERACTIONS

In this section, we present a detailed analysis of the optical backaction acting between a pair of mechanical modes coupled to a common cavity. We start from the two equations of motion

$$\ddot{z}_j = -\Omega_{{\rm m},j}^2 z - \gamma_j \dot{z}_j + f_{{\rm opt},j} \tag{S35}$$

for the normalized displacements $z_j = x_j/x_{{\rm zpf},j}$ of the two resonators $j = 1, 2$. We write the normalized optical force, arising for now from a single spring laser, as

$$f_{{\rm opt},j} = \frac{\hbar G n_{\rm c}}{m_j x_{{\rm zpf},j}} = 2\Omega_{{\rm m},j} g_{0,j} n_{\rm c}, \tag{S36}$$

where $n_{\rm c}$ is total intracavity photon number. This is given by $n_{\rm c} = n_{\max} h(u_0 + \delta u)$, where, importantly, the normalized optomechanical shift in the cavity frequency

$$\delta u = \frac{2}{\kappa} \sum_j g_{0,j} z_j \tag{S37}$$

now depends on the displacement z_j of both resonators.

We first consider driving by a single spring laser tuned to either of the maximal spring shift points $u_0 = u_\pm = \pm 1/\sqrt{3}$. Expanding $n_{\rm c}$ to third order in δu then yields

$$\begin{aligned} n_{\rm c} &= n_{\max} \left[h(u_\pm) + h'(u_\pm)\delta u + \frac{1}{6} h'''(u_\pm)(\delta u)^3 \right] && \text{(S38)} \\ &= n_{\max} \left[h(u_\pm) + h'(u_\pm) \left(\delta u - \frac{3}{4}(\delta u)^3 \right) \right], && \text{(S39)} \end{aligned}$$

where we have used $h''(u_\pm) = 0$ and $h'''(u_\pm) = -(9/2)h'(u_\pm)$. We plug this into the equation of motions to obtain

$$\begin{aligned} \ddot{z}_j = &- \Omega_{{\rm m},j}^2 z - \gamma_j \dot{z}_j + 2\Omega_{{\rm m},j} g_{0,j} && \text{(S40)} \\ &\times n_{\max} \left[h(u_\pm) + h'(u_\pm) \left(\delta u - \frac{3}{4}(\delta u)^3 \right) \right]. && \text{(S41)} \end{aligned}$$

Again, we remove the static radiation pressure contribution by redefining the displacement origin and get

$$\begin{aligned} \ddot{z}_j = &- \Omega_{{\rm m},j}^2 z - \gamma_j \dot{z}_j + 2\Omega_{{\rm m},j} g_{0,j} \\ &\times n_{\max} h'(u_\pm) \left(\delta u - \frac{3}{4}(\delta u)^3 \right). \end{aligned} \tag{S42}$$

We analyse the optical force term linear in δu,

$$\begin{aligned} f_{{\rm opt},1}^{(1)} &= 2\Omega_{{\rm m},1} \frac{2 g_{0,1} n_{\max} h'(u_\pm)}{\kappa} (g_{0,1} z_1 + g_{0,2} z_2) && \text{(S43)} \\ &= -2\Omega_{{\rm m},1} \delta\Omega_1 \, z_1 - 2\Omega_{{\rm m},1} \sqrt{\delta\Omega_1 \delta\Omega_2} \, z_2, && \text{(S44)} \end{aligned}$$

that acts on resonator 1; the equivalent for resonator 2 is obtained by exchanging indices $1 \leftrightarrow 2$. The first term in Eq. (S44) represents the intra-resonator optical spring $k_{{\rm opt},1} = 2 m_1 \Omega_{{\rm m},1} \delta\Omega_1$. Meanwhile, the cross-term in the optical force, expressed in physical units as

$$\begin{aligned} F_{{\rm opt},1}^{(1,\leftrightarrow)} &= m_1 x_{{\rm zpf},1} f_{{\rm opt},1}^{(1)} = -2 m_1 \frac{x_{{\rm zpf},1}}{x_{{\rm zpf},2}} \Omega_{{\rm m},1} \sqrt{\delta\Omega_1 \delta\Omega_2} \, x_2 \\ &= -\sqrt{2\Omega_{{\rm m},1} m_1 \delta\Omega_1} \sqrt{2\Omega_{{\rm m},2} m_2 \delta\Omega_2} \, x_2 \\ &= -\sqrt{k_{{\rm opt},1} k_{{\rm opt},2}} \, x_2 = -k_\leftrightarrow \, x_2 \end{aligned} \tag{S45}$$

represents an inter-resonator optical spring. This spring dynamically couples the displacements x_1, x_2 with spring constant $k_\leftrightarrow = \sqrt{k_{{\rm opt},1} k_{{\rm opt},2}}$.

For a pair of high-quality mechanical resonators separated by a large frequency difference $\Delta\Omega_{12} = \Omega_1 - \Omega_2 \gg \gamma_j$, as we have in our experiment, inducing a static cross-resonator spring $k_\leftrightarrow$ has virtually no effect on their dynamics. We can, however, stimulate frequency conversion between the two modes by modulating $k_\leftrightarrow$ at $\Delta\Omega_{12}$. In experiment, this is achieved by modulating the intensity $P_{\rm in} = P_{{\rm in},0}(1 + c_{\rm h} \cos(\Delta\Omega_{12} t))$ of the spring laser.

After absorbing the intra-resonator spring shifts into the mechanical frequencies $\Omega_j = \Omega_{{\rm m},j} + \delta\Omega_j$, neglecting the cubic term for now and removing terms that are not (mutually) resonant, we arrive at

$$\ddot{z}_1 = -\Omega_1^2 z - \gamma_1 \dot{z}_1 - 2\Omega_{{\rm m},1} c_{\rm h} \cos(\Delta\Omega_{12} t) \sqrt{\delta\Omega_1 \delta\Omega_2} \, z_2. \tag{S46}$$

For convenience, we write the two displacements as

$$z_j(t) = \mathrm{Re}[a_j(t)e^{-\mathrm{i}\Omega_j t}] = \frac{a_j(t)e^{-\mathrm{i}\Omega_j t} + a_j^*(t)e^{\mathrm{i}\Omega_j t}}{2} \tag{S47}$$

in terms of the complex amplitudes $a_j(t)$. We plug these into the equation of motion (S46), neglect the terms that scale with $\gamma_j/\Omega_j, J/\Omega_j \ll 1$ and remove the counter-rotating terms, to obtain the coupled differential equations

$$\begin{aligned} \dot{a}_1 &= -\frac{\gamma_1}{2}a_1 - \mathrm{i}\frac{c_\mathrm{h}\sqrt{\delta\Omega_1\delta\Omega_2}}{2}a_2, \\ \dot{a}_2 &= -\frac{\gamma_2}{2}a_2 - \mathrm{i}\frac{c_\mathrm{h}\sqrt{\delta\Omega_1\delta\Omega_2}}{2}a_1 \end{aligned} \tag{S48}$$

for the complex amplitudes. From these, we immediately identify the linear coupling rate

$$J = \frac{c_\mathrm{h}\sqrt{\delta\Omega_1\delta\Omega_2}}{2}. \tag{S49}$$

Next, we analyse the nonlinearity in the cross-resonator spring $k_\leftrightarrow$, induced by the cubic term $\propto (\delta u)^3$ in Eq. (S42). Before doing so, we isolate this (cross-Kerr) nonlinearity from the intra-resonator Duffing nonlinearity by driving the cavity with two spring lasers a and b at opposite detunings $u_0^a = u_- = -1/\sqrt{3}$, $u_0^b = u_+ = 1/\sqrt{3}$. Noting that the difference between u_- and u_+ is larger than any mechanical frequency, we can neglect the optical beating between the two lasers and decompose the total photon number

$$n_\mathrm{c} = n_\mathrm{c}^a + n_\mathrm{c}^b = n_\mathrm{max}^a h\left(u_- + \delta u\right) + n_\mathrm{max}^b h\left(u_+ + \delta u\right) \tag{S50}$$

into two independent populations $n_\mathrm{c}^{a,b}$, one sustained by each laser.

To induce resonant coupling between the mechanical modes, we modulate one of the laser intensities. Here we modulate the positive spring laser intensity $n_\mathrm{max}^a = n_\mathrm{max}(1+f(t))$ while keeping the negative spring laser at fixed intensity $n_\mathrm{max}^b = n_\mathrm{max}$, equal to the average n_max^a. After expanding Eq. (S50) to third order, we find that in the total photon number

$$\begin{aligned} n_\mathrm{c} &= n_\mathrm{max}2h(u_\pm) \\ &\quad + n_\mathrm{max}f(t)\left[h(u_+) + h'(u_+)\delta u + \frac{1}{6}h'''(u_+)(\delta u)^3\right] \\ &= n_\mathrm{max}2h(u_\pm) \\ &\quad + n_\mathrm{max}f(t)\left[h(u_+) + h'(u_+)\left(\delta u - \frac{3}{4}(\delta u)^3\right)\right] \end{aligned} \tag{S51}$$

the static linear and cubic spring shift terms cancel, leaving only the modulated part.

We plug this into the equation of motion, after discarding the static radiation pressure contribution. Moreover, as the modulation $f(t) = c_\mathrm{h}\cos(\Delta\Omega_{12}t)$ is not resonant with any of the mechanical frequencies Ω_1, Ω_2, we also discard the modulated direct radiation pressure contribution. We obtain

$$\begin{aligned} \ddot{z}_1 &= -\Omega_{\mathrm{m},1}^2 z_1 - \gamma_1\dot{z}_1 + 2\Omega_{\mathrm{m},1}g_{0,1} \\ &\quad \times f(t)n_\mathrm{max}h'(u_\pm)\delta u\left[1 - \frac{3}{4}(\delta u)^2\right] \\ &= -\Omega_{\mathrm{m},1}^2 z_1 - \gamma_1\dot{z}_1 - 2\Omega_{\mathrm{m},1}f(t)\frac{2g_{0,1}n_\mathrm{max}h'(u_\pm)}{\kappa} \\ &\quad \times [g_{0,1}z_1 + g_{0,2}z_2]\left(1 - \frac{3}{4}(\delta u)^2\right) \end{aligned}$$

and the equivalent for z_2 after exchanging indices $1 \leftrightarrow 2$. This can be conveniently expressed in terms of the linear spring shifts as

$$\begin{aligned} \ddot{z}_1 &= -\Omega_{\mathrm{m},1}^2 z_1 - \gamma_1\dot{z}_1 - 2\Omega_{\mathrm{m},1}f(t)\sqrt{\delta\Omega_1} \\ &\quad \times \Bigg[\sqrt{\delta\Omega_1}z_1\left(1 - \frac{3}{\kappa^2}\left(g_{0,1}z_1 + g_{0,2}z_2\right)^2\right) \\ &\qquad + \sqrt{\delta\Omega_2}z_2\left(1 - \frac{3}{\kappa^2}(g_{0,1}z_1 + g_{0,2}z_2)^2\right)\Bigg] \end{aligned} \tag{S52}$$

Again, we express the two displacements in terms of the complex amplitudes $a_j(t)$, employing the computer algebra software `Mathematica` to plug these into the coupled nonlinear equation of motion. After neglecting counter-rotating terms and terms scaling with $\gamma_j/\Omega_j, J/\Omega_j \ll 1$, we obtain the dynamical equations

$$\begin{aligned} \dot{a}_1 &= -\frac{\gamma_1}{2}a_1 \\ &\quad - \mathrm{i}Ja_2(t)\left[1 - \frac{9}{4}\left(\frac{g_{0,1}^2}{\kappa^2}|a_1(t)|^2 + \frac{g_{0,2}^2}{\kappa^2}|a_2(t)|^2\right)\right] \\ &\quad + \mathrm{i}Ja_1(t)\left[\frac{9}{2}\frac{g_{0,1}^2}{\kappa^2}\mathrm{Re}[a_1^*a_2]\right], \\ \dot{a}_2 &= -\frac{\gamma_2}{2}a_2 \\ &\quad - \mathrm{i}Ja_1(t)\left[1 - \frac{9}{4}\left(\frac{g_{0,1}^2}{\kappa^2}|a_1(t)|^2 + \frac{g_{0,2}^2}{\kappa^2}|a_2t)|^2\right)\right] \\ &\quad + \mathrm{i}Ja_2(t)\left[\frac{9}{2}\frac{g_{0,2}^2}{\kappa^2}\mathrm{Re}[a_2^*a_1]\right] \end{aligned} \tag{S53}$$

for the complex amplitudes. Importantly, in each second term we recognize a coupling rate

$$J_\mathrm{NL} = J\left[1 - \frac{9}{4}\left(\frac{g_{0,1}^2}{\kappa^2}|a_1(t)|^2 + \frac{g_{0,2}^2}{\kappa^2}|a_2(t)|^2\right)\right] \tag{S54}$$

that includes a nonlinear correction depending on the squared amplitudes $|a_1(t)|^2$ and $|a_1(t)|^2$. This relation is given in the main text as Eq. (9).

We initialize the nonlinear ringdown experiment in Fig. 4 with excitation in one mode only. The complex

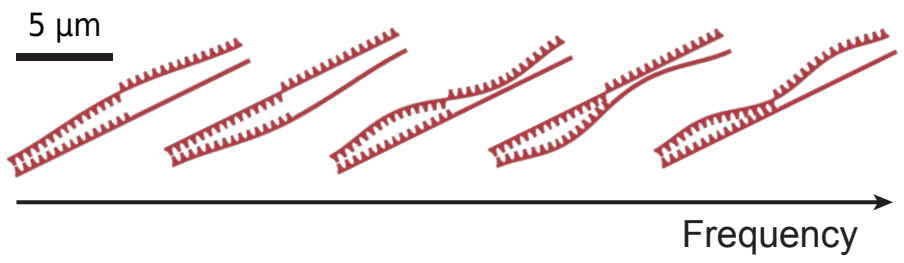

FIG. S2. **Nanobeam flexural modes.** Simulated modeshapes for the first five flexural mechanical modes. All experiments in this work employed the fourth mode, with the mode coupling experiments additionally employing the third mode.

amplitude $a_1(t)$ and $a_2(t)$ then remain $\pm\pi/2$ out of phase throughout, so that the correlation $\mathrm{Re}[a_2^* a_1] = 0$ vanishes. The third term in each equation (S53) is thus identically zero, leaving us only with a nonlinearly shifted coupling rate J_{NL}.

V. SIMULATING GEOMETRIC NONLINEARITY

We estimate the intrinsic geometric nonlinearity from finite element modelling in `COMSOL Multiphysics`. To do so, we first simulate the linear modeshape of the relevant flexural eigenmode of the device geometry, as shown in Fig. S2. We then perform a series of stationary analyses that include geometric nonlinearity. In this sweep, we impose the linear modeshape scaled by an increasing amplitude factor on the device structure, while for every amplitude, we integrate the total potential energy stored in the strain of the nanobeam [7]. This results in a potential energy $U(x) = \frac{1}{2}k_1 x^2 + \frac{1}{4}k_3 x^4$ that is quadratic-quartic in the mode displacement x, where k_1 is the linear spring constant and k_3 the coefficient for the cubic restoring force.

We fit the potential $U(x)$ obtained from the simulations to extract k_1 and k_3. We verify that k_1, when combined with the effective mass m_{eff} obtained from the same simulation, produces the correct eigenmode frequency $\Omega_{\mathrm{m}} = \sqrt{k_1/m_{\mathrm{eff}}}$. We convert k_3 into a cubic coefficient using the relation

$$\beta = \frac{k_3}{m_{\mathrm{eff}}} x_{\mathrm{zpf}}^2 \tag{S55}$$

to produce an estimated value of $\beta \approx 300$ Hz^2.

VI. EXPERIMENTAL METHODS

The experimental set-up, methods, and device employed here have been described in detail in refs. [8–10]. We briefly summarize the relevant aspects below.

A. Optomechanical system

The nano-optomechanical device under study comprised a suspended, sliced silicon nanobeam hosting a photonic crystal cavity and fabricated in-house using the methods described in [8]. The optical cavity coupled simultaneously to several non-degenerate flexural modes of the beam, whose spatial profiles are shown in Fig. S2. All experiments in this work employed the fourth mode, with the mode coupling experiments additionally employing the third mode.

Optical access was provided by free-space coupling, achieved by focusing a laser at normal incidence onto the device. Direct reflections were suppressed and cavity-emitted light was collected using a cross-polarized detection scheme. The device was mounted inside a room-temperature vacuum chamber (pressure 2×10^{-6} mbar) and positioned at the laser focus using a piezo-actuated precision stage. All quoted optical and mechanical parameters were characterized following the methods described in [8].

B. Excitation and readout

Spring shifts were induced by pumping the cavity with a strong ‘spring’ laser (Toptica CTL 1500/1550). Depending on the experiment, a secondary control laser was either used as a ‘force’ laser, or as an additional, opposing ‘spring’ laser. When used as a weak force laser, it was tuned to the cavity resonance with its intensity modulated to provide radiation pressure driving of the mechanical resonator. When used as an additional spring laser, its power was matched to the primary spring laser but operated at the opposite detuning to cancel the (nonlinear) spring effect. In that case, coherent radiation pressure driving was provided by weakly modulating either of the spring lasers instead. In the multimode coupling experiments, the intensity of a spring laser was modulated at the difference frequency of the resonator pair.

Electronic signals to drive or couple mechanical modes were generated by a high-frequency lock-in amplifier (LIA; Zurich Instruments UHFLI), amplified (Mini-Circuits ZHL-32A+ with 9 dB attenuation) and imprinted on the appropriate laser using fiber coupled electro-optic modulators (Thorlabs LN81S-FC and Covega Mach-10 056)

The instantaneous displacement $x_j(t)$ of both mechanical resonators was transduced onto the reflected intensity of light from a tertiary ‘detect’ laser (Toptica CTL 1550, incident power $P_{\mathrm{det}} = 1$ mW), far detuned from the cavity resonance ($\Delta_{\mathrm{det}} = -2.5\kappa,$). The reflected detection light was spectrally isolated from control laser light using a tunable band-pass filter (DiCon) before being directed onto a fast, AC-coupled photodiode (New Focus 1811). To extract individual resonator signals, the photodiode output was demodulated by the LIA with a 3-dB-bandwidth of 100 kHz (third order filter). The re-

sulting complex amplitudes $\tilde{a}_j(t)$ were normalized to the detector voltage level corresponding to each resonator's zero-point motion x_{zpf}, as obtained from thermally driven reference measurements without any modulations.

To account for nonlinear transduction of resonator motion onto the reflected intensity arising from the Lorentzian cavity response [6], we constructed a calibration map between the true and apparent motion amplitudes. Specifically, we numerically computed the forward map $(a_1, a_2) \to (\tilde{a}_1, \tilde{a}_2)$, where $a_j(t)$ denotes the true amplitude of sinusoidal motion of mode j, and $\tilde{a}_j$ is the corresponding apparent amplitude extracted from the cavity-transduced signal as the magnitude of the first harmonic. We then inverted this map and approximated the inverse relation $(\tilde{a}_1, \tilde{a}_2) \to (a_1, a_2)$ by fitting a fifth-order bivariate polynomial to the numerical data. This polynomial calibration was applied to the raw amplitudes reported by the LIA to recover true resonator motion amplitudes.

For time-resolved ring-down measurements, mechanical driving and coupling signals were continuously output by the LIA on separate output channels, which were then routed through individual radio frequency (RF) switches (Mini-Circuits ZYSWA-2-50DR+). An additional signal generator (Siglent SDG series) controlled the switches and triggered LIA data acquisition.

VII. EXTRACTING INSTANTANEOUS OSCILLATION FREQUENCIES

The complex resonator amplitude $a(t) = A(t)e^{\mathrm{i}\phi(t)}/x_{\mathrm{zpf}}$ reported by the LIA encodes the amplitude $A(t)$ of resonator motion, as well as its phase relative to the LIA's electronic local oscillator (LO). The demodulated phase evolves as

$$\phi(t) = \int_{t_0}^{t} [\Omega(\tau) - \omega_{\mathrm{d}}]\, \mathrm{d}\tau \tag{S56}$$

where $\Omega(t)$ is the instantaneous frequency of the resonator's motion, ω_{d} the LO frequency and t_0 the time the measurement was started. By taking the derivative of Eq. (S56), the instantaneous frequency is extracted as $\Omega(t) = \dot{\phi}(t) + \omega_{\mathrm{d}}$. This expression was evaluated for the experimental data to obtain the frequency traces shown in Fig. 1f, bottom.

VIII. ESTIMATING INSTANTANEOUS COUPLING RATES

In this section, we introduce a numerical method to estimate the instantaneous coupling $J_{\mathrm{NL}}(t)$ from the experimentally obtained complex amplitudes $a_j(t)$ of the coupled resonator pair.

The starting point is the coupled system of differential equations

$$\begin{aligned} \dot{a}_1 &= -\frac{\gamma_1}{2}a_1 - \mathrm{i}J_{\mathrm{NL}}(t)a_2, \\ \dot{a}_2 &= -\frac{\gamma_2}{2}a_2 - \mathrm{i}J_{\mathrm{NL}}(t)a_1 \end{aligned} \tag{S57}$$

that describes the evolution of a pair of resonators resonantly coupled by an effective instantaneous rate $J_{\mathrm{NL}}(t)$ (cf. Eq. (S48)). From these equations of motion, we calculate the 'excess change' in each resonator's complex amplitude as

$$\begin{aligned} \dot{a}_1 + \frac{\gamma_1}{2}a_1 &= -\mathrm{i}J_{\mathrm{NL}}(t)a_2 \\ \dot{a}_2 + \frac{\gamma_1}{2}a_2 &= -\mathrm{i}J_{\mathrm{NL}}(t)a_1. \end{aligned} \tag{S58}$$

Here, the left hand sides express the portion of the amplitude change $\dot{a}_j$ that is not attributed to the dissipation $\gamma_1 a_1/2$. From these, an estimate for $J_{\mathrm{NL}}(t)$ can be immediately obtained through dividing by $a_{2,1}$. However, these amplitudes will periodically tend to zero, giving imprecise results at those times when applied to real data.

To solve this, we square both and take the sum

$$\left|\dot{a}_1 + \frac{\gamma_1}{2}a_1\right|^2 + \left|\dot{a}_2 + \frac{\gamma_1}{2}a_2\right|^2 = J_{\mathrm{NL}}(t)^2\left(|a_1|^2 + |a_2|^2\right) \tag{S59}$$

The total energy $|a_1|^2 + |a_2|^2$ does not vanish periodically as the resonators exchange energy, so that $J_{\mathrm{NL}}(t)^2$ can be estimated reliably during the entire ringdown.

A. Correcting for local oscillator detuning

The above analysis assumes that the LIA's electronic LO frequency is tuned perfectly to the resonator. In practice, a small detuning δ_j may exist between each resonator j and its LO. This detuning appears in the demodulated signals

$$b_1(t) = c_1 e^{-\mathrm{i}\delta_1 t} a_1(t), \tag{S60}$$

$$b_2(t) = c_2 e^{-\mathrm{i}\delta_2 t} a_2(t), \tag{S61}$$

$$\tag{S62}$$

in addition to individual phase shifts $c_j = e^{-\mathrm{i}\theta_j}$. Time derivatives $\dot{a}_j$ and $\dot{b}_j$ are related through

$$\dot{a}_j(t) = c_1^* e^{\mathrm{i}\delta_j t}\left[\dot{b}_j(t) + \mathrm{i}\delta_j b_j(t)\right]. \tag{S63}$$

Plugging this into Eq. (S58) shows that the excess change in b_j,

$$\begin{aligned} c_1^* e^{\mathrm{i}\delta_1 t}\left[\dot{b}_1 + \frac{\gamma_1}{2}b_1\right] &= -\mathrm{i}J_{\mathrm{NL}}(t)a_2 - \mathrm{i}\delta_1 a_1, \\ c_2^* e^{\mathrm{i}\delta_2 t}\left[\dot{b}_2 + \frac{\gamma_2}{2}b_2\right] &= -\mathrm{i}J_{\mathrm{NL}}(t)a_1 - \mathrm{i}\delta_2 a_2, \end{aligned} \tag{S64}$$

picks up an extra contribution from the detuning.

These equations are valid for any initial condition $\{a_1(0), a_2(0)\}$. We now specialise to the situation in the experiment, where one resonator is initialized to a large amplitude $a_1(0) = a_0$, while the other starts out at rest $a_2(0) = 0$. It can be shown that, when evolving under Eqs. (S57), amplitudes $a_1(t)$ and $a_2(t)$ then remain $\pm\pi/2$ out of phase for all times $t > 0$. Consequently, the two terms in the right hand sides of Eqs. (S64) are orthogonal in the complex plane, so that the squared absolute value of their sum is the sum of their squared absolute values,

$$\begin{aligned} \left|\dot{b}_1 + \frac{\gamma_1}{2} b_1\right|^2 &= J_{\mathrm{NL}}(t)^2 |a_2|^2 + \delta_1^2 |a_1|^2, \\ \left|\dot{b}_2 + \frac{\gamma_2}{2} b_2\right|^2 &= J_{\mathrm{NL}}(t)^2 |a_1|^2 + \delta_2^2 |a_2|^2. \end{aligned} \tag{S65}$$

Noting that $|a_j| = |b_j|$, we find that naively extracting $J_{\mathrm{NL}}(t)$ from the sum of the squared excess changes

$$\begin{aligned} &\left|\dot{b}_1 + \frac{\gamma_1}{2} b_1\right|^2 + \left|\dot{b}_2 + \frac{\gamma_2}{2} b_2\right|^2 \\ &\quad = J_{\mathrm{NL}}(t)^2 \left(|b_1|^2 + |b_2|^2\right) + \delta_1^2 |b_1|^2 + \delta_2^2 |b_2|^2, \end{aligned} \tag{S66}$$

overestimates the instantaneous coupling.

In principle, this can be corrected for by evaluating

$$\begin{aligned} \dot{b}_j b_j^* &= c_j e^{-\mathrm{i}\delta_j t} \left(\dot{a}_j - \mathrm{i}\delta_j a_j\right) \times c_j^* e^{\mathrm{i}\delta_j t} a_j^* \\ &= \dot{a}_j a_j^* - \mathrm{i}\delta_j |a_j|^2. \end{aligned} \tag{S67}$$

For evolution under Eqs. (S57) starting from the experimental initial condition of single-resonator excitation, the first term is always real. We thus find the convenient relation

$$\mathrm{Im}\left[\dot{b}_j b_j^*\right] = -\delta_j |b_j|^2. \tag{S68}$$

Unfortunately, both sides of this equation again vanish periodically as the resonators exchange energy, precluding a reliable estimate of δ_j at those times. Instead, we correct for δ_j by first defining

$$\tilde{b}_j(t) = d_1^* e^{\mathrm{i}\tilde{\delta}_j} b_j(t) \tag{S69}$$

for each resonator, where d_1^* is a phase factor and $\tilde{\delta}_j$ is a correcting detuning. We then numerically optimize these parameters so that $\tilde{b}_j$ is mostly real, by minimizing

$$\min_{d_1, \tilde{\delta}_j} \int_0^T \frac{\mathrm{Im}\left[\tilde{b}_j(t)\right]^2}{|\tilde{b}_1(t)|^2 + |\tilde{b}_2(t)|^2} \, \mathrm{d}t \tag{S70}$$

over each experimental trace. Here, dividing by the total squared amplitude $|\tilde{b}_1(t)|^2 + |\tilde{b}_2(t)|^2$ ensures that the fit is not dominated by the high-amplitude segment at the start of the ringdown. At the optimal $d_1, \tilde{\delta}_j$, we then find $\tilde{b}_j \approx e^{\mathrm{i}\theta_j} a_j$ up to a phase offset θ_j, so that evaluating Eq. (S59) for $a_j \to \tilde{b}_j$ provides an unbiased estimate of the instantaneous coupling rate $J_{\mathrm{NL}}(t)$.

B. Relative amplitude correction

After extracting instantaneous coupling rates following the procedure outlined above, we observe modulations in $J_{\mathrm{NL}}(t)$ that coincide with the energy exchange between the resonators. While Eq. (S54) predicts such modulations for $g_{0,1} \neq g_{0,2}$ at high amplitude, the modulations we observe are particularly prominent at low amplitudes, where the nonlinearity should be negligible. Instead, we attribute the modulations to an error in the relative calibrations of the two amplitudes a_1 and a_2 that, in turn, affects the extracted J_{NL}. Applying a correction of $\pm 8\%$ on the two resonator amplitudes, respectively, minimizes modulations and results in instantaneous coupling rates that asymptote to the expected linear value J.

IX. SIMULATING COUPLED RESONATORS WITH FULL LORENTZIAN CAVITY RESPONSE

To validate Eq. (S54) and assess the influence of higher-order nonlinearities, we simulate the coupled dynamics of our two mechanical resonators under the full Lorentzian optical cavity response, when driven by two spring lasers. In particular, we numerically integrate the system

$$\begin{aligned} \ddot{z}_1 &= -\Omega_{\mathrm{m},1}^2 z_1 - \gamma_1 \dot{z}_1 + 2\Omega_{\mathrm{m},1} g_{0,1} \left(n_c^a + n_c^b\right), \\ \ddot{z}_2 &= -\Omega_{\mathrm{m},2}^2 z_2 - \gamma_2 \dot{z}_2 + 2\Omega_{\mathrm{m},2} g_{0,2} \left(n_c^a + n_c^b\right), \end{aligned} \tag{S71}$$

where the two laser-induced cavity populations

$$\begin{aligned} n_c^{a,b} &= n_{\max}^{a,b}(t)\, h\left(u_{a,b} + \delta u\right), \\ \delta u &= \frac{2g_{0,1}}{\kappa} z_1 + \frac{2g_{0,2}}{\kappa} z_2, \end{aligned} \tag{S72}$$

depend on the full Lorenztian $h(u)$. We accommodate for the coupling modulation with a time-dependent $n_{\max}^a(t)$ on one of the spring lasers.

To account for the shift in equilibrium positions z_j^{eq} from static radiation pressure, and the consequent shift in effective spring laser detuning $u_{a,b}^{\mathrm{eff}} = u_{a,b} + \delta u(z_1^{\mathrm{eq}}, z_2^{\mathrm{eq}})$, we numerically find a fixed-point solution for $\{z_1^{\mathrm{eq}}, z_2^{\mathrm{eq}}, u_a, u_b\}$ where $u_{a,b}^{\mathrm{eff}} = \pm 1/\sqrt{3}$ equals the two target detunings at which the linear spring shift is largest. After integration, we demodulate the displacements $z_j(t)$ to obtain complex resonator amplitudes $a_j(t)$. These are equivalent to the ones obtained in the experiment, so that we can apply the same analysis to extract instantaneous coupling rates J_{NL}.

In Fig. S3a we show coupled dynamics after initializing resonator 2 to a range of amplitudes a_0, where loss rates γ_j have been set to zero. As expected, the effective coupling rate J_{NL}, extracted from the resonator energy exchange, decreases with larger amplitude. In addition, at higher amplitudes, modulations are found in the effective coupling rate J_{NL} that do not correlate with Δ_{OM}^2. This is not predicted by Eq. (S54), and points to the influence of higher-order non-linearities.

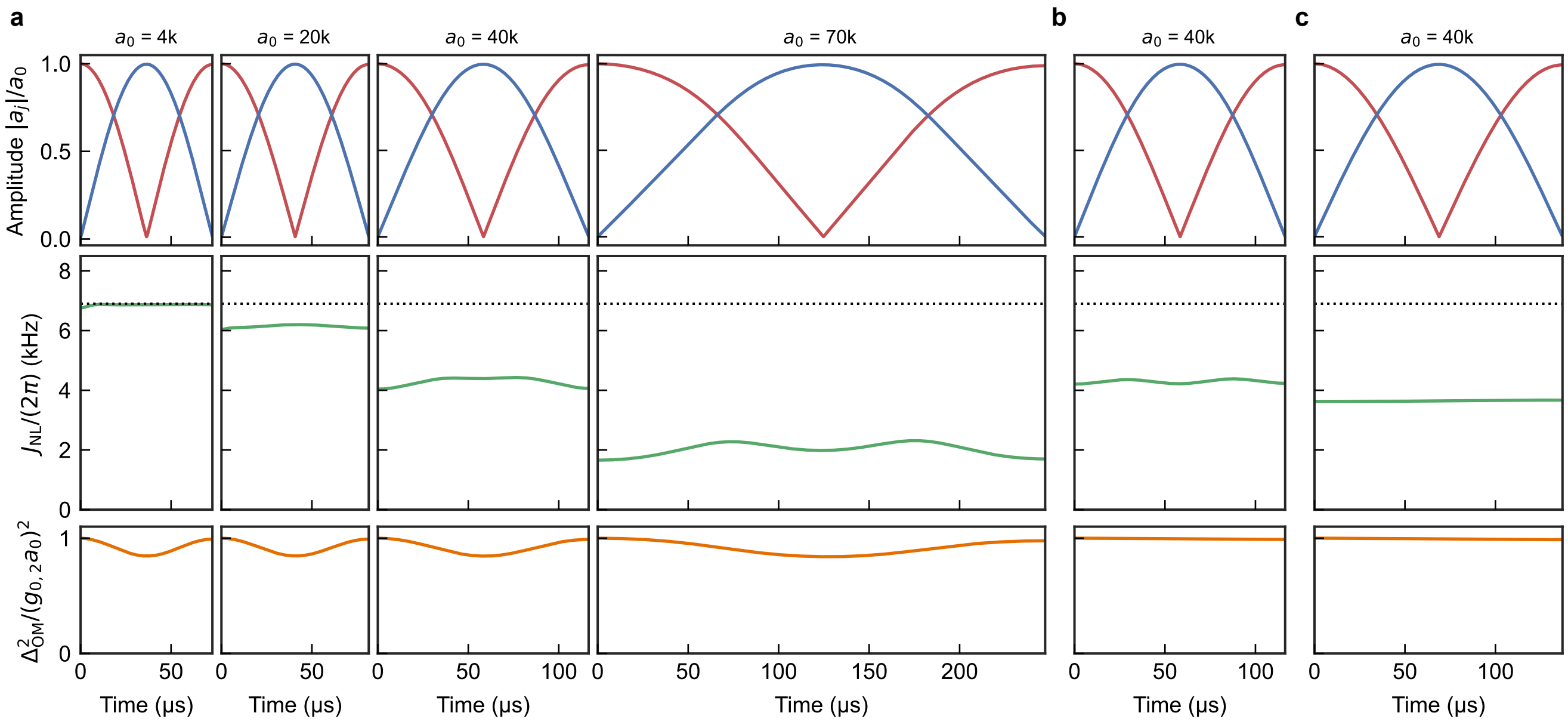

FIG. S3. **Simulated dynamics of two coupled resonators** (a) Dynamics under the full Lorentzian optical cavity response for a range of initial amplitudes a_0, accounting for the distinct values of $g_{0,j}$ found in the experiment. Loss rates γ_j are set to zero. Amplitude (top row) is exchanged between resonators 1 (blue) and 2 (red). At higher amplitudes, modulations are found in the effective coupling rate J_{NL} (middle row) that do not correlate with Δ^2_{OM} (bottom row). (b) Same, but setting $g_{0,j}$ for both resonators to the average value. Modulations in J_{NL} persists even though Δ^2_{OM} is now completely flat. (b) Same, but replacing the cavity Lorentzian by its third order approximation. The coupling rate J_{NL} is now free of modulations, indicating their higher-order origin.

To isolate these, we first run the simulation again with equal $g_{0,j}$ for both resonators (Fig. S3b). Even though Δ^2_{OM} is completely flat for this lossless scenario, the modulations in J_{NL} are seen to persist. Only when the Lorentzian cavity response $h(u)$ is replaced by its third-order Taylor expansion about each spring laser detuning do the modulations vanish (Fig. S3c), confirming their higher-order origin.

To extract the simulation curve shown in Fig. 4d of the main text, we repeat the simulations in Fig. S3a over a dense sweep of initial amplitudes a_0. For each a_0, we record the minimum and maximum values of J_{NL} and the corresponding Δ^2_{OM}. The resulting spread is shown as the grey shaded region in Fig. 4d. Notably, at low amplitudes, the simulation curve agrees with Eq. (S54).

---

[1] M. Aspelmeyer, T. J. Kippenberg, and F. Marquardt, Cavity optomechanics, Rev. Mod. Phys. **86**, 1391 (2014).

[2] B. D. Hauer, T. J. Clark, P. H. Kim, C. Doolin, and J. P. Davis, Dueling dynamical backaction in a cryogenic optomechanical cavity, Physical Review A **99**, 053803 (2019).

[3] M. Krack and J. Gross, *Harmonic balance for nonlinear vibration problems*, Mathematical Engineering (Springer International Publishing, Cham, 2019).

[4] D. W. Jordan and P. Smith, *Nonlinear ordinary differential equations: An introduction for scientists and engineers*, 4th ed. (New York : Oxford University Press, Oxford [England], 2007).

[5] J. S. Huber, G. Rastelli, M. J. Seitner, J. Kölbl, W. Belzig, M. I. Dykman, and E. M. Weig, Spectral evidence of squeezing of a weakly damped driven nanomechanical mode, Phys. Rev. X **10**, 021066 (2020).

[6] R. Leijssen, G. R. La Gala, L. Freisem, J. T. Muhonen, and E. Verhagen, Nonlinear cavity optomechanics with nanomechanical thermal fluctuations, Nat. Commun. **8**, 16024 (2017).

[7] T. M. F. Hirsch, N. P. Mauranyapin, E. Romero, G. I. Harris, X. Jin, N. Arora, C. J. Bekker, C. Meng, W. P. Bowen, and C. G. Baker, Tutorial: Membrane phononic integrated circuits, J. of Appl. Phys. **139**, 081102 (2026).

[8] J. del Pino, J. J. Slim, and E. Verhagen, Non-Hermitian chiral phononics through optomechanically induced squeezing, Nature **606**, 82 (2022).

[9] C. C. Wanjura, J. J. Slim, J. del Pino, M. Brunelli, E. Verhagen, and A. Nunnenkamp, Quadrature nonreciprocity in bosonic networks without breaking time-reversal symmetry, Nat. Phys. **19**, 1429 (2023).

[10] J. J. Slim, C. C. Wanjura, M. Brunelli, J. Del Pino, A. Nunnenkamp, and E. Verhagen, Optomechanical realization of the bosonic Kitaev chain, Nature **627**, 767 (2024).
